# The impact of recipient choice on aid effectiveness

**DOI:** 10.1016/j.worlddev.2018.10.010

**Published:** 2019-04

**Authors:** Jeremy Shapiro

**Affiliations:** Busara Center for Behavioral Economics, Kenya

**Keywords:** Cash transfers, Willingness to pay, Preferences, Poverty

## Abstract

This study compares the impact of several common development programs (agricultural extension, subsidized agricultural inputs and poultry transfers) to cash transfers equal to the cost of each program. Prior to program delivery, recipients were asked their valuation for each program (i.e., their cash indifference point between cash and the program) as a proxy for their preference between cash and the program. Subsequently, recipients were randomly assigned to receive cash or a program. Six months after delivery of cash and programs, we do not find that individuals who receive the intervention they value most are different from others in terms of consumption, food security, assets, psychological well-being or feelings of autonomy, and can rule out effects of any meaningful size. When comparing cash transfers directly to common development programs, the point estimates indicate no difference in impacts and confidence intervals rule out large differences. We do find that cash transfers increase feelings of autonomy and respect compared to non-cash interventions.

## Highlights

• Providing aid recipient’s their most valued intervention doesn’t improve effectiveness of the intervention.
• Cash transfers equal to the cost of common development programs have the same impact as the programs.
• Cash transfers increase feelings of autonomy and respect compared to non-cash interventions.

## 1 Introduction

In the past three decades, cash transfer programs have spread across countries and continents to reach an estimated 700 million people (Evans, 2017). These programs serve a variety of goals, including increasing human capital, providing emergency assistance and furnishing a general social safety net. The rise of cash transfer programs has been driven by a combination of philosophical beliefs, political economics and the perception that cash transfers are attractive from a cost-benefit perspective. In recent years multiple countries have turned to cash transfers as a general form of social protection and as a means to provide targeted assistance to vulnerable groups (Bank, 2018). Similarly, a variety of international non-governmental organizations have begun to deploy cash transfers in humanitarian aid (Hidrobo, Hoddinott, Peterman, Margolies, & Moreira, 2014a). The increasing deployment of cash transfers, as well as the research that has accompanied that trend, has led some prominent individuals and institutions in international development to suggest substituting cash transfers for other forms of aid. For example, India shifted its large cooking fuel subsidy program to a direct cash benefit transfer (Barnwal, 2014; Mittal, Mukherjee, & Gelb, 2017). Some have also suggested a cash transfer would be a more efficient program than India’s massive food transfer program (Times of India, 2013^1^). The Center for Global Development and The Gates Foundation have suggested the potential for cash transfers to replace fuel subsidies, and perhaps other commodity subsidies as well.^2^

The deployment of cash transfers at all represents a monumental change in the philosophy of aid. Whereas aid has historically focused on meeting needs of the poor as perceived by the aid community, cash transfers enable aid recipients to meet needs as perceived by themselves. This change belatedly mirrors a shift in the theoretical and philosophical underpinnings of international aid: from paternalistic colonial origins to a focus on the poor as agents in bringing about economic development. Extending cash transfers as substitutes for traditional forms of aid, such as commodity grants and subsidies, would be an act of faith in the “poor but efficient” hypothesis, pioneered by Schultz et al. (1964). Schultz posited that traditional agricultural households, a category describing a large portion of the world’s poor, make efficient allocation decisions about the resources available to them, assuming that the factors of production and technology are relatively constant. According to that hypothesis a cash transfer in lieu of commodity subsidies and various traditional forms of aid would enable recipients to efficiently allocate the cash to procure what is needed, whether that be food to enable their own labor, fertilizer, or hired skilled and unskilled labor.

The poor but efficient hypothesis, however, is not broadly accepted. Some opponents of cash transfers retain the view that the poor will misuse or squander cash, although the literature has generally allayed that concern. In particular, the literature shows that transfers do not result in unwanted impacts such as increased spending on alcohol or tobacco (Evans & Popova, 2017) and reduced labor supply (Izmalkov, 2004), and that cash transfers have positive impacts on a wide variety of welfare indicators, such as consumption, assets and educational status. The literature has firmly established that cash transfers have significant benefits and limited costs (Bastagli et al., 2016), partially legitimizing the poor but efficient hypothesis. In addition, though Schultz’s hypothesis was highly influential (Nerlove, 1999, Abler and Sukhatme, 2006), some researchers contend that empirical data is inconsistent with efficient allocation of resources by agricultural households, or holds only for specific forms of efficiency (Ball and Pounder, 1996, Shapiro, 1983, Nyariki, 2011, Umar, 2014).

Finally, the application of behavioral economics to development has suggested that behavioral biases may lead to irrational decision making, and perhaps an inefficient allocation of resources. The view put forth is not that the poor are intrinsically less rational than the rich, but that they are equally irrational, and that the condition of poverty may augment the negative effects of irrational decisions (Bertrand et al., 2004, Bernheim et al., 2015, Mani et al., 2013). The behavioral economics view has spawned a new class of behaviorally informed development interventions (e.g., Ashraf et al., 2006, Duflo et al., 2011, Duflo and Hanna, 2005). In light of this research, the behavioralist view of aid suggests that interventions should be designed to shape the decisions of aid recipients to generate optimal long-term outcomes, and cash transfers fully at the disposal of recipients may not generate these optimal outcomes.

While behavioral interventions in development have become more common, and though traditional commodity based assistance continues, the enthusiasm of the aid industry for cash transfers appears to have grown in recent years. The mutual existence of these approaches to aid creates a tension between the behavioralist and Schultzian view: are development outcomes best achieved by enabling aid recipients to optimize according to their unique information and constraints? Or is it better for the aid industry to implicitly or explicitly influence the decisions of recipients and the use of aid resources?

This study addresses this question empirically by assessing whether individuals who express a high valuation for a particular assistance program benefit more from that program than similar individuals who receive an equally valuable resource. Through a randomized controlled trial, low-income Kenyans were selected to receive either a particular non-cash development program (agricultural extension, agricultural inputs, poultry transfers) or an amount of cash equal to the cost of the program. Prior to receiving any intervention, we elicited respondents’ indifference point between cash and the program in question. Subsequently, we randomly assigned individuals to receive a program or a cash transfer equal to its cost. This design allows us to assess whether individuals who receive the intervention (cash or program) they value more, which is a proxy for their preferences, benefit differentially from the intervention, as would be expected according to the poor but efficient hypothesis. Further, by comparing cash transfers directly to various alternative interventions, we can assess whether constraining the choices of aid recipients to “spend” resources on common development interventions outperforms simple transfers, as might be expected according to the behavioral view.

Utilizing a variety of proxies for recipient preference, there is no discernible relationship between whether a recipient receives their preferred intervention (a program or cash) and components of well-being including consumption, food security, assets, psychological well-being and feelings of autonomy. When comparing cash transfers equal to the cost of common poverty reduction programs to the programs themselves, on average and irrespective of the recipient’s preference (inferred by valuation) for cash or the program, we can rule out large differences in impacts on economic outcomes. Based on 95% confidence intervals, cash transfer recipients consume no more than ∼4.25 USD more per person per month (∼10% of mean consumption) than program recipients and score no higher than 0.07 standard deviations on an index of food security than program recipients. Program recipients have no more than ∼$25 USD (8% of mean assets) than cash transfer recipients. Regardless of recipients’ valuation for specific interventions, we do find that cash transfers increase feelings of autonomy and produce more favorable views of the implementing organization than non-cash interventions. Cash transfer recipients score 0.13 standard deviations (CI = 0.05 to 0.20 standard deviations) on an index of autonomy related questions. They are more likely to believe they are trusted by the implementing NGO, that the aid they received was tailored to their needs and that they were treated as an individual.

In sum it does not appear that utilizing recipients’ stated valuation for programs, as a proxy for preferences, to allocate aid dollars between cash and programs improves the impact of aid resources. Nor do the findings imply that constraining the choices of aid recipients by delivering goods and services directly results in dramatically improved returns on aid dollars. Simply providing cash, however, does appear to improve recipients’ subjective experience of aid, without reducing the poverty reducing impact of such aid.

In addition to exploring the empirical implications of a rational/efficient view of poverty and a behavioral one, this study contributes to two additional branches of the literature: one contrasting cash transfers with other programs and the other exploring the relationship between preferences and outcomes. While, to our knowledge, there are no studies that evaluate whether incorporating recipient preferences in the allocation of resources alters the welfare impacts of aid spending, there is an emerging literature comparing cash transfers to alternative uses of aid dollars. Hidrobo, Hoddinott, Peterman, Margolies, and Moreira (2014b) compare cash transfers, food vouchers and in-kind food transfers in Ecuador, Uganda and Yemen, finding little impact of different modalities on food consumption overall but some impact on the composition of the basket consumed. Cunha (2014) finds similar results comparing cash to food transfers in Mexico. Other research contrasts studies evaluating the impacts of specific interventions with separate studies assessing the impact of cash transfers. Buera, Kaboski, and Shin (2016), for example, summarize the impacts of studies pertaining to cash transfers to micro-entrepreneurs, the impact of “graduation programs” targeting the very poor and micro-finance programs. Similarly a CGAP study compares studies evaluating the impacts of livelihood development programs, graduation programs and cash transfer programs (Sulaiman, Goldberg, Karlan, & de Montesquiou, 2016). Blattman, Jamison, and Sheridan (2017) compare cash transfers and cognitive behavioral therapy in terms of ability to reduce future crime and violence. Though illuminating, these studies are hindered by limited comparability across individual studies – each is conducted at separate times, in separate geographies using distinct measurement techniques and evaluating delivery by different implementing organizations. Thus it is difficult to make precise comparisons of the impact of cash transfers vs. alternative poverty reduction programs. This study provides a controlled comparison of cash transfers vs. several common aid programs.

Another strand of literature assesses how individual preferences, expressed by valuations, affects outcomes. This is relevant in that cash may be a more effective mode of resource delivery than alternative interventions because aid recipients have private information about what is most likely to reduce poverty. A farmer, for instance, may know her skill and whether subsidized inputs will be of any use. Jack (2013), for example, shows that landholders induced to reveal their private valuation for a tree-growing contract are more likely to produce surviving trees than randomly selected landholders. Similarly, Berry, Fischer, and Guiteras (2015) show that individuals with high willingness to pay for water filters experience greater benefits from acquisition of the filter. Additionally, a variety of studies have explored the ability to use elicited demand or subsidies as a mechanism for targeting (Cohen et al., 2015, Johnson and Lipscomb, 2017, Lybbert et al., 2018). This study builds on related literature by precisely holding constant the value of the transfer to the recipient, while exploring the relationship between recipient valuation and outcomes. Studies concerned with targeting focus on the efficiency loss of improper targeting, for example providing subsidies to individuals who are likely to purchase a good regardless of the subsidy. By holding constant the value of resources transferred, this study allows us to answer the question of whether valuation-based targeting will lead to improved outcomes. While Berry et al. (2015) can compare the outcomes for individuals with similar valuations for a water filter who either did or did not purchase the filter, this does not hold all else constant in that individuals had to pay for the filter. Thus an individual who values the filter highly and can purchase it for a (randomly) low price receives an implicit transfer which a similar individual not given the chance to purchase the filter does not receive. Jack (2013) finds that individuals who self-select into a contract to grow trees based on a lower willingness-to-accept for the contract perform better than randomly selected individuals, but this design does not enable direct comparison between individuals with the same willingness-to-accept who either do or do not receive the contract. It is possible that willingness-to-accept correlates with another variable determining response to any economic opportunity. In contrast, this study considers individuals with the same value for different interventions, who receive a transfer of precisely the same value. The studies mentioned above address the question of which households should be allocated a scarce resource (subsidized water filters or other goods, contracts to grow trees, etc.), whereas this study addresses the question of which form of aid, holding cost constant, will maximally benefit a recipient.

## 2 Study design

### 2.1 Location selection

Our goal in location selection was to identify a low-income Kenyan population, who either receive or are similar to recipients of aid. Beginning with a list of Kenyan counties, we filtered all counties with less than a 40% poverty rate, or just below the national rate of 46% (World Bank, 2015). Due to logistical considerations, we then filtered out counties with household density below the 33rd percentile. Remaining counties were then prioritized based on further proxies for poverty (fertilizer use, HIV, diarrhea and malaria prevalence, bed net use and secondary school enrollment rates). All data used comes from Kenya Open Data.^3^ Ultimately, we chose to work in Makueni county, specifically in the regions of Mbooni and Kilungu.

### 2.2 Program selection

Our aim in program selection was not to identify the “most important” aid program, but to assess whether recipient preferences are associated with outcomes across a variety of common large-scale aid programs. Detailed aid spending by specific program (e.g., agricultural extension, vaccinations) is rare, disbursed and incomplete. Thus we took a multi-step process to select programs for this study. First, we identified entities primarily responsible for funding and/or delivery of development programs in Kenya, specifically the Government of Kenya, official development assistance by multilateral and bilateral donors, philanthropic foundations and international non-governmental organization. For each of these entities we collected data on development program spending in Kenya and identified priority sectors based on the share of budget allocated to each sector. Based on this analysis, important sectors include: education, health, agriculture, water and sanitation, and humanitarian, emergency and disaster assistance (see Shapiro, 2017 for details).

From these sectors, we chose to focus on agriculture for three reasons. First, a majority of the world’s poor live in rural areas where agriculture represents a significant part of the local economy. Second, because agricultural development spending is significant in Kenya (e.g., GoK budgets ∼$700 million for agriculture programs annually). Third, in contrast to important sectors such as health and education, we expect to observe impacts of agricultural interventions within the time frame of this study.

Even within sector, it remains difficult to identify line-item expenditures or operational details for specific aid interventions. Aside from high level expenditure aggregates, detailed information on development activities is usually spelled out in project plans and reports (e.g., pertaining to the use of large scale ODA). We reviewed 8 such documents pertaining to large-scale agricultural programs (budget >$5 million) in Kenya. Of these programs, the most commonly mentioned specific intervention was agricultural extension (in 6 of 8 programs). Several programs mentioned the provision of inputs to farmers, including water, soil inputs and capital. One prominent program (the Kenya Cereal Enhancement Programme) provides a package of inputs (seeds, fertilizer, pesticides, etc.) directly to farmers. Other specific programs mentioned are livelihood development and diversification, and market linkages. We thus chose to include extension, inputs and livelihood development (poultry) in the study. These programs are suitable for the study in that they can be replicated by an NGO, are likely to have short run impacts, and are relevant in the Kenyan context as well as globally as agricultural extension and fertilizer subsides are common across the developing world.

Program details include:

1. Agricultural extension: we hired a team of 11 agriculture experts, with a combined experience of 66 years in the agricultural sector, to deliver in-person group training to randomly selected farming households. The training sessions ran from September to October 2016 – leading up to the “short rains” agricultural season in Kenya. The training included education on: land preparation, planting, soil fertility, crop selection, soil and water management, field management (fertilization, pest and disease management, weeding), record keeping and financial management, farmer group dynamics and conflict resolution, harvesting, post-harvest management, value addition and marketing.
2. Agricultural inputs: based on the advice of agricultural experts, we provided recipients with enough inputs to plant approximately 0.5 acres of cabbages or maize. The type of inputs to be provided were recommended by our agricultural consultants who determined the requirements based on terrain and crops grown in the study areas. Specifically, for the cabbage-growing region of Mbooni, we provided 50 grams of Baraka F1 seeds and 75 kg of planting fertilizer. For the maize-growing region of Kilungu, we provided 4 kg of Duma 43 seeds, 25 kg of planting fertilizer and 25 kg of top-dressing fertilizer. These inputs are roughly modeled after the Government of Kenya’s National Accelerated Agriculture Inputs Access Program. The program includes a voucher, valued at USD 60–80, to cover the cost of 10 kg of hybrid maize seed, 50 kg of basal fertilizer, and 50 kg of top-dressing fertilizer, inputs sufficient for approximately 1 acre (0.4 ha) of maize. Our agricultural inputs package also included a one-time information session on proper input usage provided by our extension agents.
3. Poultry transfers: recipients received 25 one-month old chicks vaccinated for common diseases as well as a starter pack of feed (∼10 kg). Recipients were also provided with basic information about taking care of their chicks by our team of agricultural experts and were visited occasionally by the agriculture team over the following 4 months.
4. Cash transfers: some households were randomly selected to receive direct cash transfers. The size of these transfers match the per-recipient cost of one of the above programs – $15 for agricultural extension, $75 for agricultural inputs in Mbooni and $35 for agricultural inputs in Kilungu, and $120 for poultry transfers. These transfers account for the direct value of items transferred as well overhead costs, however we ignore any implicit labor costs (e.g., labor that is complimentary to the program delivered or the use of the cash). Cash transfers were delivered using the M-Pesa mobile money platform.

### 2.3 Baseline survey

Eligible individuals comprised those over 18 years of age residing in a home made of all or partially natural materials (e.g., wood, local stone or mud, excluding homes which include cement or cinder blocks) and with relatively small land holdings (less than 6 hectares). We surveyed 3008 individuals meeting these criteria. Each respondent was administered a baseline survey that elicited their indifference point between cash and the relevant programs (agricultural extension, agricultural inputs or poultry transfer). We chose a valuation based approach to estimate whether a respondent prefers cash or the program, as opposed to a direct choice between the two. This choice was made as a valuation approach could potentially be extended to multiple interventions of various costs (if preferences matter, it may be wise to provide the program with the highest ratio of valuation to cost). To obtain a valuation, we simply asked what amount of cash, if given directly to the respondent, would make them as well off as receiving the intervention. A prior exercise investigating various methods to obtain valuations suggested this method was as reliable as various other common valuation techniques, such at the Becker-DeGroot-Marschakh method and multiple price lists (Jang & Shapiro, 2018). The survey also measured a variety of baseline characteristics. The survey was administered on tablet computers using SurveyCTO software. The baseline survey was conducted from August 10th, 2016 to September 24th, 2016.

Data integrity was maintained through continuous monitoring of data coming into the server to check for missing observations and inconsistencies in responses, back check surveys to establish the reliability of data, GPS checks to establish the presence of a structure at the recorded interview site and random spot checks of interviews.

### 2.4 Randomization

Study participants were randomly assigned to receive a particular non-cash program or a cash transfer equal to the cost of the program. As described above, the interventions differed by study location on account of agricultural characteristics. Study participants in Kilungu were randomly assigned to receive agricultural extension, a transfer of $14, inputs for maize or a transfer of $35. Study participants in Mbooni were randomly assigned to receive agricultural inputs for cabbage, a transfer of $75, 25 month-old chicks or a transfer of $120. Randomization was conducted at the individual level. Though the informational components of extension may have spillover effects, individual randomization is a deliberate choice: the primary goal of this study is to compare across cash or program arms in order to isolate the effect of the choice mechanism. In equilibrium, were recipients to be given a choice between programs and cash transfers, we expect some would choose the program, thus having a mix of those receiving cash and the program in the same village provides the most relevant comparison.

### 2.5 Intervention implementation

For respondents receiving a non-cash intervention, the goods or services were delivered in person by an individual not involved in the initial data collection. At that visit, the respondent’s name, ID number and location were verified. In the event of discrepancies, the program delivery was delayed until further investigation. For respondents receiving cash, a transfer was sent through the M-Pesa digital payment platform. This platform allowed the researchers to confirm the name from the survey matches the name associated with the mobile money account. Finally, we followed up with a sample of recipients (by phone or in person) to confirm receipt of goods, services or cash.

1. Agricultural extension: An agricultural training curriculum consisting of 6 sessions was developed by contracted agricultural consultants. These sessions were administered by our team of agriculture experts at a location convenient for the respondents to attend. Respondents randomly selected to receive this intervention were contacted via phone for identity verification and invited to attend training sessions at a nearby venue on specific dates. A few farmers who could not come to the training venue received training on their farms instead. Out of 500 respondents, 431 attended these training sessions.
2. Agricultural inputs: Inputs were procured from a well-known seed distributor in Nairobi and transported to the target areas by the supplier. Before the goods were disbursed, participants were contacted via phone and all identity and contact information provided at baseline was verified. After verification, respondents were contacted via phone and informed of the inputs collection point and were advised and encouraged to collect their inputs. 280 respondents in Mbooni and 179 in Kilungu collected their inputs, meaning 459 of 500 recipients collected the inputs.
3. Poultry transfers: The chicks were procured from a well-known seed and livestock distributor in Nairobi and transported to the target areas by the supplier. Before the goods were disbursed, respondents were contacted via phone and advised to construct suitable chicken coops in preparation for the storage of the birds. This call was made two weeks before the chicks were scheduled to be collected by the respondents. A week after the first preparation call, respondents were again contacted via phone and reminded to construct suitable chicken coops if they had not yet done so. They were also advised on suitable storage conditions for the chicks. After the preparation reminder calls, respondents were contacted via phone and informed of a date and venue to collect the chicks. At collections, respondents were advised on basic upkeep and care. 489 out of 505 respondents showed up to collect their chicks. Agricultural trainers visited the chick recipients on a rotating basis to answer any queries and assist with care for the chicks.
4. Cash transfers: As cash transfers were to be implemented through M-Pesa, respondents’ M-Pesa numbers were verified before the transfer was initiated. Additionally, the name of the respondent was matched with the name under which the M-Pesa account was registered before the transfer was initiated. All respondents scheduled to receive cash transfers were contacted on the phone to be informed of the impending transfer and the amount. Out of targeted respondents in each category, 491 respondents received the agricultural extension equivalent cash transfer, 483 received the agricultural inputs equivalent cash transfer, and 497 received the poultry equivalent cash transfer. The remaining respondents refused the cash transfer when contacted.

### 2.6 Endline survey and outcomes

The endline survey was conducted from April 6th, 2017 to June 10th, 2017, or approximately 6 months after the intervention. This timing was selected by our agricultural team, to coincide with the harvest time (allowing time for sale) and when chicks were sufficiently mature to sell. We chose this time horizon because it is when individuals receiving programs will reap the maximal benefit in terms of the outcomes considered here (e.g., consumption, food security, subjective well-being). Moreover, cash transfers have been shown to have measurable impacts at time-horizons as short as 6 to 9 months (Blattman et al., 2013, Fafchamps et al., 2011, Haushofer and Shapiro, 2016) thus we also expect to detect impacts of cash transfers at this time horizon.

Our primary outcomes of interest for this study are:

1. Consumption – total monthly per capita consumption, including the value of own production, in Kenyan shillings. Consumption is winsorized at the 99th percentile.
2. Food security – weighted standardized index.
3. Assets – total value of household assets, excluding land and buildings, in Kenyan shillings. Assets are winsorized at the 99th percentile and the 1st percentile (due to negative outliers occurring due to household debt).
4. Psychological well-being – weighted standardized index.
5. Autonomy, dignity, trust – weighted standardized index.

In constructing weighted standardized indices we follow Anderson (2008). For a group of related outcomes, we first calculate the co-variance matrix. We then invert the matrix and define weights for each variable as the row sums of the inverted co-variance matrix. Related outcomes are then de-meaned and divided by the standard deviation of one treatment group (cash recipients). The index is constructed as the weighted sum of standardized outcomes, and is finally re-centered by the mean and standard deviation of the index for the cash recipient group. When estimating each of the equations below for these primary outcomes, we adjust *p*-values based on 5 outcomes of interest and report Family Wise Error Rate adjustments.

### 2.7 Design summary

The figure below provides an overview of the study, including: baseline dates, number of respondents allocated to each treatment group at baseline, number of respondents actually receiving each treatment, number of respondents reached at endline.

**Figure:**
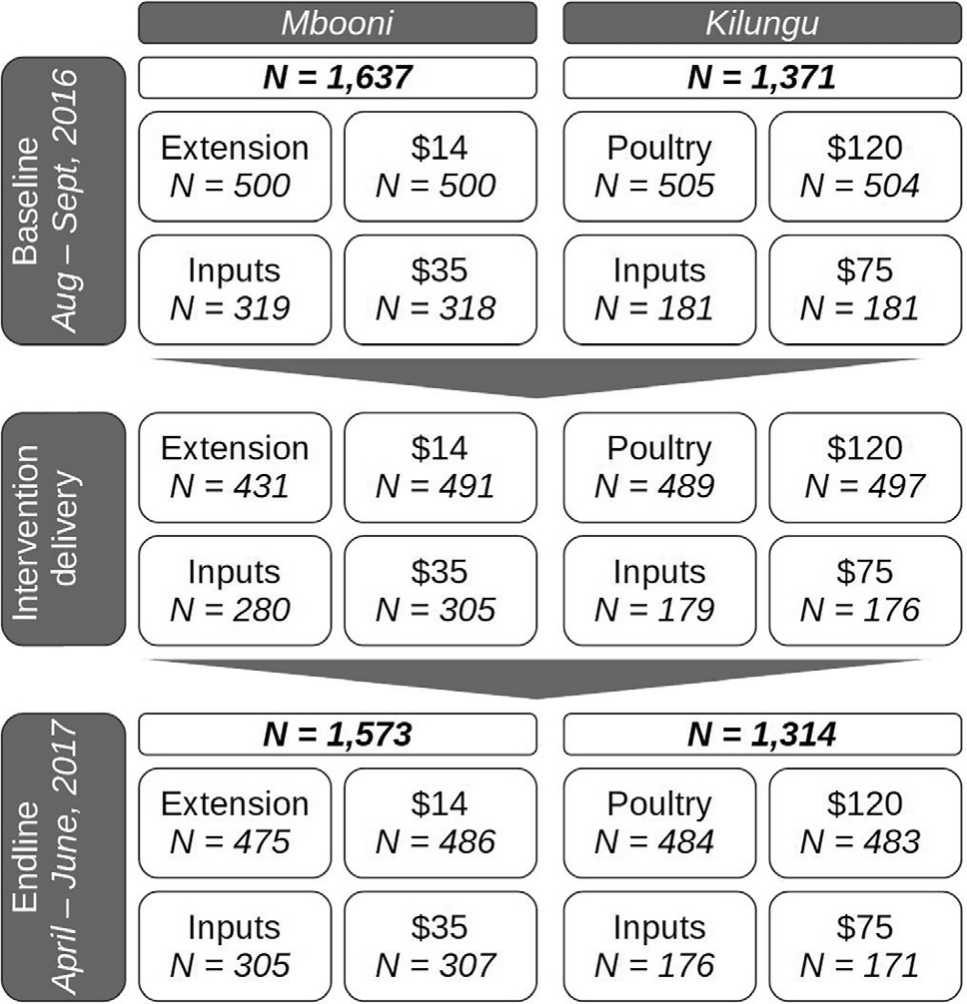

## 3 Results

The aim of this study is to understand whether recipient preferences over various aid programs impact outcomes, while holding the value of aid received constant. To minimize the chance that particular program characteristics drive results, we chose to implement three programs. In the analysis, we pool data across the three programs and use fixed effect to control for the aggregate value of the resources received by the recipient (whether cash or non-cash goods and services). Note the pooled sample remains balanced according to the (randomly determined) proportion who receive either cash or a program and whether an individual receives cash or a program is uncorrelated with sample characteristics. This analysis is similar to Banerjee et al. (2015), who pool data from several related, but operationally distinct Graduation programs.

The analysis below was pre-registered with the AEA RCT Registry (AEARCTR-0002015) on February 23, 2017, two months before the start of endline data collection. Deviations from the pre-analysis plan are described in footnotes below, and all analyses specified in the pre-analysis plan, including results by individual program, are presented in the Online appendix.

### 3.1 Sample characteristics and balance

Of the initial 3008 respondents, we resurveyed 2887 or a re-contact rate of 96%. Though small overall, we also confirm that attrition is not correlated with treatment status: attrition is 3.6% in the cash recipient group and 4.2% in the program recipient group (*p* = 0.41). In Table 1 we show the means of various baseline characteristics in the estimation (endline) sample by intervention group. The table confirms that treatment assignment is not correlated with observed baseline characteristics in the estimation sample, and thus should not be correlated with the error term in endline specifications. Further, the table indicates that differential attrition based on baseline covariates did not occur. Of 27 comparisons, we observe two differences significant at the 5% level and one difference significant at the 10% level.

**Table 1:** Baseline balance across extension, inputs and poultry recipients at endline.

	(1) Cash Extension	(2) Extension	p-value: (1) = (2)	N	(3) Cash Inputs	(4) Inputs	p-value: (3) = (4)	N	(5) Cash Poultry	(6) Poultry	p-value: (5) = (6)	N
Age of respondent	44.397	43.288	0.267	961	43.567	44.420	0.377	959	43.598	43.370	0.817	967
	(0.698)	(0.714)			(0.672)	(0.694)			(0.700)	(0.696)		
Gender of respondent (dummy = 1 if female)	0.570	0.623	0.093^∗^	961	0.636	0.624	0.694	959	0.619	0.624	0.875	967
	(0.022)	(0.022)			(0.022)	(0.022)			(0.022)	(0.022)		
Psychological wellbeing index (CESD, GHQ-12, WVS)	−0.003	0.032	0.590	961	0.060	−0.007	0.287	959	−0.019	−0.063	0.502	967
	(0.046)	(0.046)			(0.044)	(0.045)			(0.046)	(0.047)		
Growth mindset	31.932	31.878	0.911	961	32.745	31.927	0.101	959	32.230	32.343	0.816	967
	(0.343)	(0.341)			(0.350)	(0.355)			(0.349)	(0.337)		
Grit	3.381	3.410	0.368	961	3.385	3.454	0.038^∗∗^	959	3.380	3.395	0.643	967
	(0.022)	(0.023)			(0.023)	(0.024)			(0.023)	(0.023)		
Valuation for Extension (in 10,000 KES)	2.765	2.993	0.496	961	2.378	2.571	0.520	959	2.179	2.097	0.693	967
	(0.226)	(0.247)			(0.202)	(0.223)			(0.151)	(0.142)		
Valuation for Inputs (in 10,000 KES)	1.993	1.994	0.992	961	1.741	1.725	0.911	959	1.510	1.384	0.155	967
	(0.112)	(0.123)			(0.102)	(0.099)			(0.063)	(0.062)		
Valuation for Poultry (in 10,000 KES)	4.023	4.170	0.619	961	3.662	3.627	0.898	959	2.774	2.602	0.367	967
	(0.200)	(0.216)			(0.198)	(0.195)			(0.136)	(0.133)		
Valuation for intervention allocated (in 10,000 KES)	2.765	2.993	0.496	961	1.741	1.725	0.911	959	2.774	2.602	0.367	967
	(0.226)	(0.247)			(0.102)	(0.099)			(0.136)	(0.133)		
Respondent preferences respected (based on cost)	0.035	0.954	0.000^∗∗∗^	961	0.224	0.784	0.000^∗∗∗^	959	0.356	0.624	0.000^∗∗∗^	967
	(0.008)	(0.010)			(0.019)	(0.019)			(0.022)	(0.022)		
Number of household members	5.202	5.032	0.255	961	4.937	5.164	0.154	959	4.950	4.884	0.669	967
	(0.102)	(0.108)			(0.116)	(0.110)			(0.106)	(0.112)		
Share of household expenditure on food in past week	0.646	0.667	0.150	961	0.644	0.646	0.866	959	0.666	0.646	0.161	967
	(0.010)	(0.010)			(0.010)	(0.010)			(0.010)	(0.010)		
Household wealth (in 10,000 KES)	85.709	75.503	0.232	961	63.190	79.111	0.029^∗∗^	959	53.965	48.437	0.306	967
	(6.490)	(5.542)			(4.442)	(5.757)			(3.899)	(3.737)		

*Notes:* Table shows balance of mean of variables measured at baseline which are listed on the left across all baseline recipients allocated to receive extension, inputs or poultry, or a cash equivalent. Respondent’s valuation for interventions and household wealth are top-coded at 99th percentile. Standard errors are in parenthesis. ^∗^ p < 0.1, ^∗∗^ p < 0.05, ^∗∗∗^ p < 0.01.

The table also shows summary statistics related to the valuations respondents ascribe to the various programs in question. On average, respondents value agricultural extension at KES 29100 (the average among the three intervention groups varies between ~20000 and 30000), with a median value of KES 10000, relative to an estimated cost of KES 1500. Poultry is valued, on average, at KES 38800, with a median of KES 20000, compared to a cost of KES 12000. In Kilungi, inputs are valued on average at KES 15100, with a median of KES 10000, relative to a cost of KES 3700, and at KES 23000 in Mbooni, also with a median of 10000, relative to a cost of KES 7600. As would be expected based on the relatively high valuations ascribed to the programs, a higher percentage of individuals randomly assigned to receive the program instead of cash get their more valued intervention. In the extension intervention group, 95% of those receiving the program got their more highly valued intervention compared to 4% among those randomly assigned to receive cash. In the inputs intervention group, 78% of those that received inputs valued them more than cash, while only 22% of those that received cash valued the cash more than the inputs. In the poultry group, 62% in the group receiving poultry value that more than the cash, while 36% of those receiving cash value the cash more than the poultry.

Since valuation is not randomly assigned, we explore what characteristics are correlated with the valuation placed on each program. In Table 2 we regress recipient valuation for each program on a set of individual and household characteristics. Household wealth is positively correlated with valuation, which may be the result of wealthier households being more able to generate economic returns from programs or simply that wealthier households have a higher willingness to pay for programs. Households with a higher expenditure on food value programs less than others, which may be the result of having a higher desire for liquid cash to meet immediate needs. Women value programs significantly less than men. The age of a respondent, which may be a proxy for experience farming and raising livestock, is negatively correlated with valuations. Given that most valuations are significantly higher than the costs of the programs, this may reflect the effect of experience causing individuals to have valuations of programs that are more in line with the cost of providing those programs. Finally, individuals scoring higher on the psychological Grit scale have significantly higher valuation, this may reflect optimism about the respondent’s ability to generate returns from the program. The valuation placed on a program by the respondent may be an arbitrarily complex function of demographic characteristics and psychological attributes, and we are not able to fully map out that function. The implication is that in the analysis below, we take the valuation as a meaningful variable in its own right, in that inquiring about valuation may allow programs to be better targeted, but do not investigate what may underlie the effects related to valuation.

**Table 2:** Determinates of valuation.

	(1) Extension	(2) Inputs	(3) Poultry
Age of respondent	−449	−581^∗∗∗^	−557^∗^
	(288)	(136)	(263)
Age – squared	4	5^∗∗∗^	5^∗^
	(3)	(1)	(3)
Gender of respondent (dummy = 1 if female)	−6541^∗∗∗^	−1986^∗^	−3601^∗^
	(1757)	(832)	(1604)
Psychological wellbeing index (CESD, GHQ-12, WVS)	1674	718	777
	(863)	(409)	(788)
Growth mindset	161	105^∗^	−402^∗∗∗^
	(110)	(52)	(101)
Grit	7104^∗∗∗^	4364^∗∗∗^	4431^∗∗^
	(1704)	(807)	(1556)
Number of household members	263	645^∗∗∗^	761^∗^
	(357)	(169)	(326)
Share of household expenditure on food in past week	411	−3563^∗^	−6899^∗^
	(3669)	(1739)	(3351)
Household wealth (in 10,000 KES)	25^∗∗∗^	16^∗∗∗^	24^∗∗∗^
	(7)	(4)	(7)
Constant	8352	12210^∗∗^	46570^∗∗∗^
	(9313)	(4413)	(8505)
			
Observations	2887	2887	2887

*Notes:* Table shows correlation between recipient valuation for each program and various characteristics. Age is in years, psychological index is mean zero with SD = 1, Growth mindset is a scale from 0 to 50, Grit is a scale from 1 to 5, and share of expenditure on food is expressed as a decimal percent. Respondent’s valuation for interventions and household wealth are top-coded at 99th percentile. Standard errors are in parenthesis. ^∗^ p < 0.1, ^∗∗^ p < 0.05, ^∗∗∗^ p < 0.01.

### 3.2 Heterogeneous impacts by recipient valuation

To answer the question of whether individuals who value a particular program benefit more from receiving that program as compared to similar individuals receiving a cash transfer, we pool data across the three interventions and estimate:

(1) $$y_{i}=\alpha_{p}+\beta_{1}{\mathrm{Program}}_{i}+\beta_{2}{\mathrm{Program}}_{i}v_{i}+\beta_{3}v_{i}+\varepsilon_{i}$$

where *v* is the ratio of the respondent’s expressed value for the program to the cost of the program for person *i*, and *Program* is an indicator that recipient *i* received a program rather than cash. $\alpha_{p}$ are dummies controlling for the aggregate value of the resources received by the recipient (cash or a program costing that cash value). The variation exploited here is the random variation, conditional on value of resources received, in whether a recipient gets cash or a non-cash intervention. Receiving a cash transfer of any value is the omitted category. In this and all specifications we use robust standard errors. If individuals who value programs much more than the cost benefit from the programs more than others, we expect that $\beta_{2}>0$. As *v* is unbounded, we assess the impact of outliers by reporting results for several transformations of *v*, including: *v* winsorized at 95th percentile, log(v) and *v* where any value-cost ratio above 10 is capped at 10.^4^

In Table 3 we present the coefficient of interest $\left(\beta_{2}\right)$ for all transformations of *v*. For the sake or readability, other terms in the regressions are omitted. The results do not indicate that recipient valuations of programs have any discernible impact on outcomes, and the 95% confidence intervals rule out any meaningful effects. With respect to consumption, column 1 suggests that, for program recipients, moving from the 25th percentile of valuation (1.25X program cost) to the 75th percentile (6.5X program value) results in an increase of KSH 13 ($0.13) in monthly consumption. Removing outliers (column 4) implies a similarly modest KSH 45 increase. Moreover, in the primary specification (column 1) the 95% confidence interval includes only changes in consumption of less than 1%. Even in the most favorable estimation (column 4) the 95% confidence interval indicates that shifting from the 25th to 75th percentile of valuation increases consumption by less than ∼9%.

**Table 3:** Effect of type of program or cash allocation on primary outcome variables.

	Program X Val-Cost Ratio	Program X Val-Cost Ratio (trimmed)	Program X ln(Val-Cost Ratio)	Program X Val-Cost Ratio (capped)	N	Mean of dependent variable
Monthly Per-Capita Cons (KES)	2.540	7.230	41.661	8.726	2887	4486.27
	(1.658)	(13.970)	(90.645)	(36.209)		
	[0.810]	[0.950]	[0.840]	[0.960]		
						
Food Security Index	0.000	−0.002	−0.032	−0.014	2887	0
	(0.001)	(0.004)	(0.025)	(0.011)		
	[0.890]	[0.950]	[0.680]	[0.640]		
						
Household Assets (KES)	30.940	181.974	1025.844	383.446	2887	34282.49
	(27.892)	(146.093)	(916.244)	(339.552)		
	[0.760]	[0.670]	[0.680]	[0.750]		
						
Psychological Wellbeing Index	0.002^∗∗∗^	0.000	0.006	0.003	2887	−.03
	(0.001)	(0.004)	(0.027)	(0.011)		
	[0.150]	[0.950]	[0.840]	[0.960]		
						
Autonomy Index	0.000	−0.005	−0.040	−0.008	2887	−.06
	(0.001)	(0.005)	(0.030)	(0.011)		
	[0.890]	[0.700]	[0.630]	[0.820]		

*Notes:* Outcome variables are listed on the left. Each cell displays the coefficient for the interaction term from a regression of the outcome variable on program assignment, variants of the valuation-cost ratio and their interaction along with intervention fixed effects. Program is a dummy which takes the value 1 if respondent received a program instead of cash. Valuation is the respondent’s stated valuation at baseline for the program into which they were randomized. Cost is estimated as the cost incurred to deliver a program per respondent. Costs are fixed for all respondents who were randomized to receive a given program or its cash equivalent in a given location. Standard errors are reported in parenthesis, FWER-adjusted p-values are reported in brackets. ^∗^p < 0.1, ^∗∗^p < 0.05, ^∗∗∗^p < 0.01.

The results are similar with respect to assets. In the primary specification (column 1), shifting from the 25th to 75th percentile of valuation for program recipients increases assets by at most 2%, according to the 95% confidence interval. The 95% confidence interval of the most favorable estimation, when top-coding the valuation cost ratio, suggests that the maximum difference in assets between those that received the program but valued it little (25th percentile) and those that valued it more (75th percentile) is 16% of assets, although the point estimate is a more modest 6% difference.

Turning to the indices, the 95% confidence intervals for the food security, psychological well-being and autonomy indices indicate that program recipients with a valuation in the 75th percentile score no higher than 0.1 standard deviations (~0.15 in the most favorable specification) on these indices than those in the 25th percentile. The point estimate in column 1 is statistically different from zero for the psychological well-being index, but the remaining columns indicate this is due to outliers. Thus it appears that the degree to which respondents value the program they receive has very little impact on outcomes. As seen in Table 2, various individual and household characteristics are correlated with valuation. It is therefore possible that one of these characteristics is driving the lack of relationship between valuation and outcomes. These results, however, are robust to controlling for the variables included in Table 2 (see Appendix).

A possible concern with this conclusion is that measurement error in recipient valuations (i.e., the recipient’s reported valuation is not precisely equal to their true valuation) would lead to attenuation bias, and an underestimate of the relationship between recipient valuation and outcomes. While possible, given the relatively small estimated magnitude of most of the coefficients, such bias would have to be severe for attenuation bias to mask an economically meaningful relationship between recipient valuations and other outcomes. Also, while such bias could cause us to erroneously conclude there is no relationship between recipient’s true preferences and outcomes (as might be predicted by economic theory), from a policy perspective (in terms of deciding how to target interventions) we can still confidently conclude that there is no relationship between outcomes and expressed valuations, since the latter relationship is estimated without bias.

As an alternative proxy for recipient preferences, we define an indicator variable *R* which is equal to 1 if the respondent values what they received more than the alternative. Specifically, *R* equals 1 if either: (a) the respondent received a cash transfer equal to the cost of the program and they value the program less than its cost or (b) the respondent received the program and they value it more than its cost. *R* is equal to 0 otherwise. We pool data across the three programs and estimate:

(2) $$y_{i}=\alpha_{p}+\beta_{1}R_{i}+\varepsilon_{i}$$

where $\alpha_{p}$ again control for the aggregate value of resources transferred.

Considering the impact of respecting a respondents’ preferences (i.e., provide cash if they value the program less than the cost and the program otherwise) Table 4 presents the results. We can rule out meaningful impacts on economic outcomes (consumption, food security and assets), however it appears providing respondents with the item they value most reduces psychological well-being by 0.07 standard deviations (not significant when correcting for multiple hypotheses) and reduces feelings of autonomy by 0.11 standard deviations. The latter result is robust to accounting for multiple hypotheses via FWER (p=0.03). This result is driven primarily by individuals receiving a program: of those who received what they most valued, 79% received a program and 21% received cash. Thus, to the extent receiving a program is correlated with receiving the more valued intervention, the former might also influence the results. When controlling for whether the individual received a program or cash, which is randomly determined, the relationship between receiving the more valued intervention no longer holds, as show in Table 5. In this table we also see a positive impact of receiving cash on our autonomy index, even when controlling for whether the respondent receives the intervention they value most.

**Table 4:** Effect of type of program or cash allocation on primary outcome variables.

	Constant	Respondent preferences respected (as per cost)	N
Monthly Per-Capita Cons (KES)	4362.703^∗∗∗^	−140.129	2887
	(117.575)	(131.417)	
		[0.580]	
			
Food Security Index	0.037	−0.041	2887
	(0.044)	(0.035)	
		[0.580]	
			
Household Assets (KES)	34991.659^∗∗∗^	−423.570	2887
	(1188.447)	(1174.823)	
		[0.720]	
			
Psychological Wellbeing Index	−0.032	−0.067^∗^	2887
	(0.036)	(0.036)	
		[0.290]	
			
Autonomy Index	−0.207^∗∗∗^	−0.111^∗∗∗^	2887
	(0.038)	(0.038)	
		[0.000]^∗∗∗^	

*Notes:* Outcome variables are listed on the left. Each cell displays the coefficient for the interaction term from a regression of the outcome variable on program assignment, variants of the valuation-cost ratio and their interaction along with intervention fixed effects. Program is a dummy which takes the value 1 if respondent received a program instead of cash. Valuation is the respondent’s stated valuation at baseline for the program into which they were randomized. Cost is estimated as the cost incurred to deliver a program per respondent. Costs are fixed for all respondents who were randomized to receive a given program or its cash equivalent in a given location. Standard errors are reported in parenthesis, FWER-adjusted p-values are reported in brackets. ^∗^p < 0.1, ^∗∗^p < 0.05, ^∗∗∗^p < 0.01.

**Table 5:** Effect of recipient preferences being respected in allocation on primary outcome variables, with controls.

	Constant	Respondent preferences respected (as per cost)	Cash	N
Monthly Per-Capita Cons (KES)	4257.767^∗∗∗^	−62.885	132.797	2887
	(179.374)	(164.139)	(164.320)	
		[0.770]	[0.800]	
				
Food Security Index	0.069	−0.065	−0.040	2887
	(0.049)	(0.040)	(0.040)	
		[0.430]	[0.800]	
				
Household Assets (KES)	35669.138^∗∗∗^	−922.266	−857.347	2887
	(1637.535)	(1411.689)	(1411.359)	
		[0.770]	[0.800]	
				
Psychological Wellbeing Index	−0.056	−0.049	0.030	2887
	(0.051)	(0.045)	(0.045)	
		[0.570]	[0.800]	
				
Autonomy Index	−0.280^∗∗∗^	−0.058	0.091^∗∗^	2887
	(0.052)	(0.046)	(0.046)	
		[0.570]	[0.150]	

*Notes:* Outcome variables are listed on the left. Regression includes intervention fixed effects with agricultural extension as omitted category. Respondent preferences respected is an indicator that takes the value 1 if either the respondent values the program less than the cost and receives cash, or the respondent values the program more than the cost and receives the program. Standard errors are reported in parenthesis, FWER-adjusted p-values are reported in brackets. ^∗^p < 0.1, ^∗∗^p < 0.05, ^∗∗∗^p < 0.01.

### 3.3 Assessing the relative impacts of constrained and unconstrained choice

While including recipient preferences in aid allocation decisions has little impact, its possible that the allocation decisions of the aid industry perform better on average than the sum of individual decisions by recipients. To assess this possibility, we estimate the impact of receiving a cash transfer compared to receiving goods and services costing an amount equal to the cash transfer:

(3) $$y_{i}=\alpha_{p}+\beta_{1}{\mathrm{Cash}}_{i}+\varepsilon_{i}$$

Table 6 shows the results from this specification. The point estimate suggests that households receiving cash transfers have monthly per capital consumption KSH 169 (∼1.5 USD) higher than those who received programs costing an equivalent amount but this difference is not significantly different from zero. Moreover, the 95% confidence interval rules out large differences in consumption among cash and program recipients – the upper bound of the interval is ∼4.25 USD which is approximately 10% of the sample mean monthly consumption. The results similarly rule out large impacts of cash on food security and assets in comparison to programs – the 95% confidence interval puts the maximum increment of cash over programs at 0.07 standard deviations for food security and negative ∼25 USD, or 8% of the sample mean for assets. With regards to psychological well-being, there are no significant differences between cash and program recipients with the 95% confidence interval ruling out differences greater than 0.13 standard deviations. Even when adjusting for multiple hypotheses, the estimates suggest that cash transfer recipients score 0.13 standard deviations higher on the autonomy index, significant at the 1% level.

**Table 6:** Effect of cash allocation on primary outcome variables.

	Constant	Cash	N
Monthly Per-Capita Cons (KES)	4208.517^∗∗∗^	169.366	2887
	(128.553)	(131.556)	
		[0.540]	
			
Food Security Index	0.018	−0.003	2887
	(0.033)	(0.035)	
		[0.950]	
			
Household Assets (KES)	34946.850^∗∗∗^	−321.023	2887
	(1235.305)	(1174.576)	
		[0.950]	
			
Psychological Wellbeing Index	−0.094^∗∗∗^	0.059	2887
	(0.036)	(0.036)	
		[0.400]	
			
Autonomy Index	−0.325^∗∗∗^	0.125^∗∗∗^	2887
	(0.040)	(0.038)	
		[0.000]^∗∗∗^	

*Notes:* Outcome variables are listed on the left. Regression includes intervention fixed effects with agricultural extension as omitted category. Cash is a dummy which takes the value 1 if respondent received a cash transfer instead of a program. Standard errors are reported in parenthesis, FWER-adjusted p-values are reported in brackets. ^∗^p < 0.1, ^∗∗^p < 0.05, ^∗∗∗^p < 0.01.

Table 7 shows the drivers of the autonomy index. The results indicate that cash transfer recipients are more likely to believe they are trusted by the implementing NGO, that the aid they received was tailored to their needs and that they were treated as an individual. They are less likely to report being treated with contempt by the implementing organization, that they were persuaded to make a particular choice and that they can ask the NGO for what they need.

**Table 7:** Effect of cash allocation on autonomy sub-outcome variables.

	Constant	Cash	N
Autonomy Index	−0.325^∗∗∗^	0.125^∗∗∗^	2887
	(0.040)	(0.038)	
		[0.000]^∗∗∗^	
I make important decisions in my life for myself	3.611^∗∗∗^	−0.014	2883
	(0.022)	(0.021)	
		[0.940]	
Other people and orgs enable me to live with dignity	3.076^∗∗∗^	−0.022	2880
	(0.037)	(0.035)	
		[0.940]	
NGOs trust the people they seek to help	3.374^∗∗∗^	0.065^∗∗^	2866
	(0.030)	(0.026)	
		[0.080]^∗^	
I would rather have little money but make my own decisions	3.534^∗∗∗^	0.026	2876
	(0.026)	(0.024)	
		[0.860]	
Org from whom I received aid treated me as an equal	3.620^∗∗∗^	0.025	2407
	(0.030)	(0.027)	
		[0.880]	
Org from whom I received aid treated me with contempt	1.655^∗∗∗^	−0.111^∗∗∗^	2412
	(0.047)	(0.038)	
		[0.020]^∗∗^	
Org from whom I received aid was arrogant	1.175^∗∗∗^	−0.006	2413
	(0.023)	(0.021)	
		[0.950]	
Aid was tailored to solve my problems	3.187^∗∗∗^	0.250^∗∗∗^	2415
	(0.035)	(0.032)	
		[0.000]^∗∗∗^	
Org from whom I received aid treated me as an individual	3.223^∗∗∗^	0.247^∗∗∗^	2411
	(0.041)	(0.032)	
		[0.000]^∗∗∗^	
Org from whom I received aid ridiculed me	0.011^∗∗∗^	−0.004	2416
	(0.004)	(0.003)	
		[0.830]	
I felt that I could ask the org for what I needed	0.380^∗∗∗^	−0.064^∗∗∗^	2416
	(0.021)	(0.019)	
		[0.000]^∗∗∗^	
Org from whom I received aid reduced my sense of control	0.213^∗∗∗^	0.004	2416
	(0.018)	(0.016)	
		[0.950]	
Org tried to persuade me to make a particular decision	0.177^∗∗∗^	−0.038^∗∗∗^	2416
	(0.017)	(0.015)	
		[0.070]^∗^	
Org made me feel in control of my life	0.774^∗∗∗^	0.020	2416
	(0.017)	(0.016)	
		[0.830]	

*Notes:* Outcome variables are listed on the left. Regression includes intervention fixed effects with agricultural extension as omitted category. Cash is a dummy which takes the value 1 if respondent received a cash transfer instead of a program. Standard errors are reported in parenthesis, FWER-adjusted p-values are reported in brackets. ^∗^p < 0.1, ^∗∗^p < 0.05, ^∗∗∗^p < 0.01.

All additional analysis discussed in the pre-analysis plan, including detailed index component results, results by program and heterogeneous results, are shown in the Online appendix.

## 4 Conclusion

Through a randomized controlled trial low-income Kenyans were randomly selected to receive either a particular development program (agricultural extension, agricultural inputs, poultry transfers) or an amount of cash equal to the cost of the program. Prior to receiving any intervention, we elicited respondents’ indifference point between cash and the program in question. Subsequently, we randomly assigned individuals to receive a program or a cash transfer equal to its cost. This design addresses two questions: first, what is the relative impact of common development programs relative to cash transfers equal to the cost of the program? Second, would incorporating recipients’ valuation for programs into the decision of how to allocate aid dollars affect the impact of aid programs?

Utilizing a variety of proxies for recipient preference, there is no discernible relationship between whether a recipient receives their more valued intervention (a program or cash) and components of well-being including consumption, food security, assets, psychological well-being and feelings of autonomy. Moreover, the 95% confidence intervals rule out effects of any meaningful magnitude. When comparing cash transfers to common aid programs we can rule out large differences in impacts on economic outcomes. Based on 95% confidence intervals, cash transfer recipients consume no more than ∼4.25 USD more per person per month (∼10% of mean consumption) than program recipients and score no higher than 0.07 standard deviations on an index of food security than program recipients. Program recipients have no more than ∼$25 USD (8% of mean assets) than cash transfer recipients. We do find that cash transfers increase feelings of autonomy and produce more favorable views of the implementing organization than non-cash interventions.

The results speak directly to the theoretical ambiguity about whether replacing traditional forms of direct aid with cash transfers will lead to optimal or sub-optimal outcomes. The Schultzian view suggests cash will be allocated efficiently by recipients, while the behavioral view suggests behavioral frictions might reduce the efficacy of cash. These results suggest that behavioralist concerns are not warranted with respect to the provision of cash transfers. Previous research has established that cash transfers do not result in increased expenditure on temptation goods like alcohol or tobacco, as might be predicted e.g., by hyperbolic discounting. This study goes a step further and suggests that other behavioral frictions do not reduce the impact of cash transfers in comparison to traditional forms of direct aid, which constrain the choices of recipients. We show that the poor are reasonably efficient in their allocation of cash resources, in the sense that they do as well as aid professionals who have indicated a tendency towards programs such as extension and inputs. Thus the results indicate that, with respect to cash transfers, the poor-but-efficient hypothesis is reasonable, and interference to constrain recipient choices is not warranted.

An important caveat is that this conclusion may not apply to all interventions and it does not contradict the behavioralist view in total, as behavioral concerns may impact domains such as health and education. Nevertheless, when aid is intended to augment the income of the poor in a domain where market imperfections are unlikely (e.g., as in the provision of subsidized agricultural inputs or knowledge) the poor make equally good use of resources as aid professionals.

From a policy perspective, the results offer two specific implications. The first is that the means of aid interventions have welfare implications in addition to the ends of those programs. While there is little gain from an impact perspective (e.g., on income) of incorporating recipient preferences into allocating aid resources, we do find that the choice enabled by cash transfers results in greater feelings of autonomy and favorable opinions of NGOs. Thus aid organizations and governments should consider a holistic view of recipient welfare, including perceptions of the process as well as “hard” economic outcomes in choosing among available interventions. Second, in the arena of the transfer of private, or even semi-private, goods and services, cash transfers are likely a dominant intervention whenever alternative programs necessitate high overheads. This study finds no difference in impact from cash transfers equal to the all-in cost of interventions (including the specific value of items transferred and delivery cost), but does not account for general administrative and overhead costs. Thus, when agencies operate programs providing goods and services such as subsidized commodities and basic agricultural extension with high overhead costs, they are likely destroying value for recipients relative to cash transfers.

## Declaration of interest

None.

## Figures and Tables

**Table 8:** Effect of type of program or cash allocation on primary outcome variables – with controls.

	Program X Val-Cost Ratio	Program X Val-Cost Ratio (trimmed)	Program X ln(Val-Cost Ratio)	Program X Val-Cost Ratio (capped)	N	Mean of dependent variable
Monthly Per-Capita Cons (KES)	4.283^∗∗∗^	13.608	65.971	14.633	2887	4486.27
	(1.437)	(11.870)	(75.432)	(28.745)		
	[0.880]	[0.940]	[0.870]	[0.980]		
						
Food Security Index	0.000	−0.003	−0.036	−0.015	2887	0
	(0.001)	(0.004)	(0.025)	(0.011)		
	[0.880]	[0.940]	[0.620]	[0.590]		
						
Household Assets (KES)	39.887	161.293	920.680	316.134	2887	34282.49
	(26.126)	(143.893)	(897.752)	(335.252)		
	[0.750]	[0.660]	[0.620]	[0.670]		
						
Psychological Wellbeing Index	0.002^∗∗∗^	−0.000	0.003	0.001	2887	−.03
	(0.001)	(0.004)	(0.028)	(0.011)		
	[0.110]	[0.940]	[0.870]	[0.980]		
						
Autonomy Index	0.000	−0.006	−0.044	−0.010	2887	−.06
	(0.001)	(0.005)	(0.030)	(0.012)		
	[0.880]	[0.710]	[0.580]	[0.890]		

*Notes:* Outcome variables are listed on the left. Each cell displays the coefficient for the interaction term from a regression of the outcome variable on program assignment, variants of the valuation-cost ratio and their interaction along with intervention fixed effects. Program is a dummy which takes the value 1 if respondent received a program instead of cash. Valuation is the respondent’s stated valuation at baseline for the program into which they were randomized. Cost is estimated as the cost incurred to deliver a program per respondent. Costs are fixed for all respondents who were randomized to receive a given program or its cash equivalent in a given location. Control variable include: Age of respondent, Age – squared, Gender, Psychological wellbeing index, Growth mindset, Grit, Number of household members, Share of household expenditure on food in past week, Household wealth. Standard errors are reported in parenthesis, FWER-adjusted p-values are reported in brackets. ^∗^ p < 0.1, ^∗∗^ p < 0.05, ^∗∗∗^ p < 0.01.

## Appendix A

### Indices and variables

Variables or indices below marked with a ${}^{+}$ are primary outcomes of interest. Variables or indices below marked with a # are secondary outcomes. Impacts on variables or indices below marked with a ^∗^ will also be reported to illuminate the specific cause of the change in the primary outcome.

1. Consumption^+^ – monthly KES consumption per capita
  (a) Food^∗^
    i. Food own production^∗^
    ii. Food bought
      A. Meat, fish & dairy^∗^
      B. Fruit & vegetables^∗^
      C. Cereals^∗^
      D. Other food^∗^
  (b) Temptation good expenditure^∗^
    i. Alcohol
    ii. Tobacco
    iii. Gambling
  (c) Airtime, internet, other phone expenses^∗^
  (d) Travel, transport, hotels^∗^
  (e) Personal and household items^∗^
    i. Clothing and shoes
    ii. Personal items such as soap, shampoo, etc.
    iii. Household items such as matches, kerosene, etc.
    iv. Cooking fuel
  (f) Recreation/entertainment^∗^
  (g) Housing^∗^
    i. Rent
    ii. Electricity
    iii. Water
  (h) Education expenditures^∗^
  (i) Medical expenditure^∗^
  (j) Social expenditure^∗^
    i. Religious expenses or other ceremonies
    ii. Weddings
    iii. Funerals
    iv. Charitable donations
    v. Dowry/bride price
    vi. Fees paid to the village elder, chiefs or other officials
  (k) Other expense greater than KSH 1000
2. Food security^+^ – weighted standardized index of:
  (a) Number of times last month adults cut or skipped meals (negatively coded)^∗^
  (b) Number of times last month children cut or skipped meals (negatively coded)^∗^
  (c) Number of times last month had to borrow food or rely on help from a friend or relative (negatively coded)^∗^
  (d) All household members eat two meals a day (indicator)^∗^
  (e) All household members usually eat until content (indicator)^∗^
  (f) Number of times last week respondent has eggs, meat or fish^∗^
3. Income^#^ – sum (KSH) of monthly household income from:
  (a) Livestock^∗^
    i. Cows
      A. Value of milk (sold and consumed)
      B. Value of meat (sold and consumed)
      C. Value of animals sold
      D. Value of other products
      E. Cost of care (e.g. fodder, veterinary care, etc.)
    ii. Small ruminants
      A. Value of meat (sold and consumed)
      B. Value of animals sold
      C. Value of other products
      D. Cost of care (e.g. fodder, veterinary care, etc.)
    iii. Birds
      A. Value of eggs (sold and consumed)
      B. Value of meat (sold and consumed)
      C. Value of animals sold
      D. Cost of care (e.g. fodder, veterinary care, etc.)
  (b) Agricultural income (monthly average)^∗^
    i. Value of crops harvested in short rains season
    ii. Costs of seeds, fertilizers/herbicides/pesticides, hired machines, water, labor and other expenses in short rains season
  (c) Enterprise income^∗^
    i. Sales in prior month (prorated for share of enterprise owned if applicable)
    ii. Costs of electricity, wages, water, transport, purchase of inputs, other costs (prorated for share of enterprise owned if applicable)
  (d) Wage income^∗^
    i. Sum of income from outside labor
4. Assets^+^ – sum (in KSH) of value of:
  (a) Productive assets^∗^
    i. Irrigation pump
    ii. Hose pipe
    iii. Ox-Ploughs
    iv. Oxen/work bulls
    v. Knapsack sprayers
    vi. Wheelbarrows
    vii. Ox-carts/donkey carts
    viii. Hand carts
    ix. Other farming tools
    x. Fishing equipment (boats, canoes, etc)
    xi. Other asset used for agriculture or business
  (b) Vehicles^∗^
    i. Bicycle
    ii. Motorbike
  (c) Furniture^∗^
    i. Sofas
    ii. Chairs
    iii. Table
    iv. Clock/Watch
    v. Beds
    vi. Mattresses
    vii. Cupboards
    viii. Other furniture
  (d) Household durables^∗^
    i. Cell phone
    ii. Sewing machine
    iii. Radio, tape- OR CD player
    iv. Battery
    v. Solar panel
    vi. Television or computer
    vii. Kerosene stove
    viii. Refrigerator
    ix. Insecticide treated bed net
  (e) Other
  (f) Livestock^∗^
    i. Cows
    ii. Birds
    iii. Small ruminants
  (g) Financial assets^∗^ – net balance (KSH) of savings minus outstanding loans:
    i. Savings with an institution (bank, SACCO, micro-finance organization)
    ii. Savings with M-Pesa
    iii. Savings in any other place (e.g., with family or friends)
    iv. Loans made by friends or family
    v. Loans from moneylenders, micro-finance institutions, shops, banks or other sources
5. Psychological well-being^+^ – weighted standardized index of:
  (a) CESD (depression)^∗^ with standard scoring ( https://www.outcometracker.org/library/CES-D.pdf)
  (b) GHQ-12^∗^ with standard scoring
  (c) WVS (happiness)^∗^ (1–4 scale)
  (d) WVS (life satisfaction)^∗^ (1–10 scale)
6. Autonomy, dignity, trust^+^ – weighted standardized index of:
  (a) “I feel that I am autonomous – I make the important decisions in my life for myself”^∗^ (1–4 scale)
  (b) “Other people and organizations enable me to live with dignity”^∗^ (1–4 scale)
  (c) “NGOs and organizations that try to lift people from poverty trust the people they seek to help”^∗^ (1–4 scale)
  (d) “I would rather have little money and make my own decisions than have more money and let others make my decisions” (1–4 scale)
  (e) “The organization and people from whom I received the aid treated me as an equal” (1–4 scale)
  (f) “The organization and people from whom I received the aid treated me with contempt” (1–4 scale)
  (g) “The organization and people from whom I received the aid behaved arrogantly” (1–4 scale)
  (h) “The aid I received was tailored for my benefit and to solve my problems” (1–4 scale)
  (i) “The organization providing the aid treated me as an individual, not just another one of the masses” (1–4 scale)
  (j) “Did anyone from the organization from whom you received the aid ridicule you?” (0–1)
  (k) “Did you feel that you could ask the person who gave you the aid for what you needed, and make demands upon them?” (0–1)
  (l) “Did the organization and people from whom you received the aid do anything to reduce your sense that you could control your own life?” (0–1)
  (m) “Did the organization and people from whom you received the aid try to persuade you to make a particular decision?” (0–1)
  (n) “Did the organization and people from whom you received the aid do anything to help you feel in control of your life?” (0–1)
7. Labor^#^ – Hours spent per week per capita on income generating activities, including:
  (a) Working in agriculture for this household^∗^
  (b) Tending animals for this household^∗^
  (c) Working in a non-farm or livestock business owned by this household^∗^
  (d) Working for pay for someone outside the household (in agriculture, livestock, housework, casual labor, salaried job or other paid work)^∗^
8. Education index^#^
  (a) Weighted standardized index of:
    i. Proportion of children (<19) in school^∗^
    ii. Average days of school missed per child (<19)^∗^ – *negatively coded*
    iii. Average perception of child (<19) school performance^∗^
    iv. Average spending on school expenses per child (<19)^∗^
    v. Average of highest level of education expect children (<19) will complete^∗^
    vi. Average time studying or in school per child (<19)^∗^
9. Sources of heterogeneity (as measured at baseline)
  (a) Age (of respondent)
  (b) Gender (indicator for female)
  (c) Wealth
    i. Land and buildings – sum (in KSH) of value of:
      A. House and the land under it
      B. Fish pond
      C. Other buildings (e.g., sheds)
      D. Land
    ii. Assets – defined above (excluding value of livestock)
  (d) Mindset
  (e) Grit

## Footnotes

1 https://timesofindia.indiatimes.com/city/pune/Cash-transfer-more-efficient-than-PDSis-efficient-way-for-extending-food-subsidy/articleshow/18096216.cms.

2 https://www.cgdev.org/sites/default/files/digital-payments-platform-state-capacity.pdf.

3 https://opendata.go.ke/dataset/Kenya-Government-Funded-Projects-2015/ncdd-s55u; https://opendata.go.ke/dataset/Kenya-Open-Budget-Program-Based-Budget-2015/5jh8-v7sc.

4 These transformations of *v* were not specified in the pre-analysis plan and were added upon observing the distribution of *v*.
