# Supplementary material for: The impact of recipient choice on aid effectiveness
Source: World Dev. 2019 Apr;116:137–49. doi: 10.1016/j.worlddev.2018.10.010 (PMC6358116; doi:10.1016/j.worlddev.2018.10.010)
Supplement: Supplementary file 1 [file mmc1.pdf]

List of Tables

1 Effect of program allocation and valuation-cost ratio on primary outcome variables 2
2 Effect of program allocation and winsorized valuation-cost ratio on primary outcome variables . . 3
3 Effect of program allocation and log of valuation-cost ratio on primary outcome variables 4
4 Effect of program allocation and capped valuation-cost ratio on primary outcome variables 5
5 Effect of program allocation and valuation on primary outcome variables for different intervention recipients . . . 6
6 Effect of recipient preferences being respected in allocation (as per median valuation) on primary outcome variables . . . 7
7 Effect of recipient preferences being respected in allocation (as per median valuation) on primary outcome variables for different intervention recipients . . . 8
8 Effect of recipient preferences being respected in allocation (as per cost) on primary outcome variables . . . 9
9 Effect of recipient preferences being respected in allocation (as per cost) on primary outcome variables for different intervention recipients . . . 10
10 Effect of cash allocation on primary outcome variables . . . 11
11 Effect of cash allocation on primary outcome variables for different intervention recipients 12
12 Effect of type of program or cash allocation on primary outcome variables . . . 13
13 Multivariate regression of primary outcome variables on indicator for recipient preferences being respected in allocation (as per median valuation) and age . . . 14
14 Multivariate regression of primary outcome variables on indicator for recipient preferences being respected in allocation (as per cost) and age . . . 15
15 Multivariate regression of primary outcome variables on indicator for recipient preferences being respected in allocation (as per median valuation) and gender . . . 16
16 Multivariate regression of primary outcome variables on indicator for recipient preferences being respected in allocation (as per cost) and gender . . . 17
17 Multivariate regression of primary outcome variables on indicator for recipient preferences being respected in allocation (as per median valuation) and wealth . . . 18
18 Multivariate regression of primary outcome variables on indicator for recipient preferences being respected in allocation (as per cost) and wealth . . . 19
19 Multivariate regression of primary outcome variables on indicator for recipient preferences being respected in allocation (as per median valuation) and growth mindset . . . 20
20 Multivariate regression of primary outcome variables on indicator for recipient preferences being respected in allocation (as per cost) and growth mindset . . . 21
21 Multivariate regression of primary outcome variables on indicator for recipient preferences being respected in allocation (as per median valuation) and grit . . . 22
22 Multivariate regression of primary outcome variables on indicator for recipient preferences being respected in allocation (as per cost) and grit . . . 23
23 Multivariate regression of primary outcome variables on indicator for cash allocation and age . . 24
24 Multivariate regression of primary outcome variables on indicator for cash allocation and gender . 25
25 Multivariate regression of primary outcome variables on indicator for cash allocation and wealth . 26
26 Multivariate regression of primary outcome variables on indicator for cash allocation and growth mindset . . . 27
27 Multivariate regression of primary outcome variables on indicator for cash allocation and grit . . 28
28 Effect of program allocation and valuation-cost ratio on secondary outcome variables 29
29 Effect of program allocation and winsorized valuation-cost ratio on secondary outcome variables . 30
30 Effect of program allocation and log of valuation-cost ratio on secondary outcome variables . . . 31
31 Effect of program allocation and capped valuation-cost ratio on secondary outcome variables . . . 32
32 Effect of program allocation and valuation on secondary outcome variables for different intervention recipients . . . 33

33 Effect of recipient preferences being respected in allocation (as per median valuation) on secondary outcome variables . . . 34
34 Effect of recipient preferences being respected in allocation (as per median valuation) on secondary outcome variables for different intervention recipients . . . 35
35 Effect of recipient preferences being respected in allocation (as per cost) on secondary outcome variables . . . 36
36 Effect of recipient preferences being respected in allocation (as per cost) on secondary outcome variables for different intervention recipients . . . 37
37 Effect of cash allocation on secondary outcome variables . . . 38
38 Effect of cash allocation on secondary outcome variables for different intervention recipients . . . 39
39 Effect of type of program or cash allocation on secondary outcome variables . . . 40
40 Multivariate regression of secondary outcome variables on indicator for recipient preferences being respected in allocation (as per median valuation) and age . . . 41
41 Multivariate regression of secondary outcome variables on indicator for recipient preferences being respected in allocation (as per cost) and age . . . 42
42 Multivariate regression of secondary outcome variables on indicator for recipient preferences being respected in allocation (as per median valuation) and gender . . . 43
43 Multivariate regression of secondary outcome variables on indicator for recipient preferences being respected in allocation (as per cost) and gender . . . 44
44 Multivariate regression of secondary outcome variables on indicator for recipient preferences being respected in allocation (as per median valuation) and wealth . . . 45
45 Multivariate regression of secondary outcome variables on indicator for recipient preferences being respected in allocation (as per cost) and wealth . . . 46
46 Multivariate regression of secondary outcome variables on indicator for recipient preferences being respected in allocation (as per median valuation) and growth mindset . . . 47
47 Multivariate regression of secondary outcome variables on indicator for recipient preferences being respected in allocation (as per cost) and growth mindset . . . 48
48 Multivariate regression of secondary outcome variables on indicator for recipient preferences being respected in allocation (as per median valuation) and grit . . . 49
49 Multivariate regression of secondary outcome variables on indicator for recipient preferences being respected in allocation (as per cost) and grit . . . 50
50 Multivariate regression of secondary outcome variables on indicator for cash allocation and age . 51
51 Multivariate regression of secondary outcome variables on indicator for cash allocation and gender 52
52 Multivariate regression of secondary outcome variables on indicator for cash allocation and wealth 53
53 Multivariate regression of secondary outcome variables on indicator for cash allocation and growth mindset . . . 54
54 Multivariate regression of secondary outcome variables on indicator for cash allocation and grit . 55
55 Effect on Consumption . . . 56
56 Effect on Food Security . . . 57
57 Effect on Household Income . . . 58
58 Effect on Household Assets . . . 59
59 Effect on Psychological Indices . . . 60
60 Effect on Autonomy . . . 61
61 Effect on Labour . . . 62
62 Effect on Education . . . 63

Table 1: Effect of program allocation and valuation-cost ratio on primary outcome variables

	Constant	Program	Valuation-cost ratio	Program X Valuation-cost ratio	N
Monthly Per-Capita Cons (KES)	4436.163***	−188.709	−35054.147**	25395.901	2887
	(125.089)	(134.900)	(16119.997)	(16583.380)	
		[0.390]	[0.730]	[0.820]	
Food Security Index	0.021	−0.002	−3.898	4.684	2887
	(0.048)	(0.036)	(5.305)	(5.359)	
		[1.000]	[0.780]	[0.820]	
Household Assets (KES)	34732.180***	17.381	−159433.562	309395.643	2887
	(1252.921)	(1199.442)	(224343.939)	(278921.710)	
		[1.000]	[0.780]	[0.800]	
Psychological Wellbeing Index	−0.012	−0.077**	−18.133***	20.575***	2887
	(0.038)	(0.037)	(6.332)	(7.049)	
		[0.150]	[0.180]	[0.120]	
Autonomy Index	−0.187***	−0.128***	−7.361	3.973	2887
	(0.041)	(0.039)	(6.993)	(9.573)	
		[0.000]***	[0.780]	[0.820]	

NOTES: Outcome variables are listed on the left. Regression includes intervention fixed effects with agricultural extension as omitted category. Program is a dummy which takes the value 1 if respondent received a program instead of cash. Valuation is the respondent's stated valuation at baseline for the program into which they were randomized. Cost is estimated as the cost incurred to deliver a program per respondent. Costs are fixed for all respondents who were randomized to receive a given program or its cash equivalent in a given location. Valuation-cost ratio is expressed per 10000 units. Standard errors are reported in parenthesis, FWER-adjusted p-values are reported in brackets. * $p < 0.1$, ** $p < 0.05$, *** $p < 0.01$

Table 2: Effect of program allocation and winsorized valuation-cost ratio on primary outcome variables

	Constant	Program	Valuation-cost ratio (trimmed)	Program X Valuation-cost ratio (trimmed)	N
Monthly Per-Capita Cons (KES)	4607.003***	–210.047	–215310.598**	72300.344	2887
	(167.844)	(164.532)	(107777.623)	(139697.660)	
		[0.380]	[0.390]	[0.950]	
Food Security Index	0.036	0.016	–11.156	–23.538	2887
	(0.064)	(0.043)	(33.266)	(38.055)	
		[0.830]	[0.910]	[0.950]	
Household Assets (KES)	35540.622***	–714.296	–1250941.387	1819738.739	2887
	(1650.149)	(1444.787)	(1047963.093)	(1460928.197)	
		[0.830]	[0.650]	[0.780]	
Psychological Wellbeing Index	–0.028	–0.061	–8.017	4.714	2887
	(0.051)	(0.045)	(37.342)	(44.568)	
		[0.380]	[0.910]	[0.950]	
Autonomy Index	–0.160***	–0.095**	–20.178	–53.694	2887
	(0.052)	(0.047)	(32.358)	(47.341)	
		[0.140]	[0.880]	[0.780]	

NOTES: Outcome variables are listed on the left. Regression includes intervention fixed effects with agricultural extension as omitted category. Program is a dummy which takes the value 1 if respondent received a program instead of cash. Valuation is the respondent's stated valuation at baseline for the program into which they were randomized. Cost is estimated as the cost incurred to deliver a program per respondent. Costs are fixed for all respondents who were randomized to receive a given program or its cash equivalent in a given location. Valuation-cost ratio is expressed per 10000 units and winsorized at the 95th percentile. Standard errors are reported in parenthesis, FWER-adjusted p-values are reported in brackets. * $p < 0.1$, ** $p < 0.05$, *** $p < 0.01$

Table 3: Effect of program allocation and log of valuation-cost ratio on primary outcome variables

	Constant	Program	ln(Valuation-cost ratio)	Program X ln(Valuation-cost ratio)	N
Monthly Per-Capita Cons (KES)	4573.631***	−214.693	−104.036	41.661	2887
	(185.206)	(172.691)	(75.825)	(90.645)	
		[0.540]	[0.610]	[0.880]	
Food Security Index	−0.000	0.036	0.016	−0.032	2887
	(0.070)	(0.044)	(0.024)	(0.025)	
		[0.600]	[0.870]	[0.610]	
Household Assets (KES)	36322.387***	−758.216	−1065.217	1025.844	2887
	(1762.774)	(1500.944)	(702.442)	(916.244)	
		[0.650]	[0.550]	[0.610]	
Psychological Wellbeing Index	−0.042	−0.064	0.002	0.006	2887
	(0.053)	(0.046)	(0.022)	(0.027)	
		[0.540]	[0.950]	[0.880]	
Autonomy Index	−0.166***	−0.085*	−0.007	−0.040	2887
	(0.057)	(0.049)	(0.023)	(0.030)	
		[0.330]	[0.950]	[0.610]	

NOTES: Outcome variables are listed on the left. Regression includes intervention fixed effects with agricultural extension as omitted category. Program is a dummy which takes the value 1 if respondent received a program instead of cash. Valuation is the respondent's stated valuation at baseline for the program into which they were randomized. Cost is estimated as the cost incurred to deliver a program per respondent. Costs are fixed for all respondents who were randomized to receive a given program or its cash equivalent in a given location. Valuation-cost ratio is expressed per 10000 units. Standard errors are reported in parenthesis, FWER-adjusted p-values are reported in brackets. * $p < 0.1$, ** $p < 0.05$, *** $p < 0.01$

Table 4: Effect of program allocation and capped valuation-cost ratio on primary outcome variables

	Constant	Program	Valuation-cost ratio (capped)	Program X Valuation-cost ratio (capped)	N
Monthly Per-Capita Cons (KES)	4579.648***	–205.301	–320320.455	87264.159	2887
	(220.502)	(205.588)	(297274.444)	(362087.676)	
		[0.620]	[0.730]	[0.970]	
Food Security Index	0.003	0.055	46.310	–137.021	2887
	(0.084)	(0.054)	(112.747)	(112.465)	
		[0.620]	[0.880]	[0.680]	
Household Assets (KES)	36883.709***	–1168.354	–4170877.592	3834458.899	2887
	(2045.559)	(1771.737)	(2732830.060)	(3395524.079)	
		[0.620]	[0.470]	[0.710]	
Psychological Wellbeing Index	–0.039	–0.068	0.218	25.386	2887
	(0.063)	(0.056)	(88.034)	(107.271)	
		[0.570]	[1.000]	[0.970]	
Autonomy Index	–0.148**	–0.095	–61.854	–80.258	2887
	(0.065)	(0.059)	(85.668)	(114.829)	
		[0.360]	[0.880]	[0.870]	

NOTES: Outcome variables are listed on the left. Regression includes intervention fixed effects with agricultural extension as omitted category. Program is a dummy which takes the value 1 if respondent received a program instead of cash. Valuation is the respondent's stated valuation at baseline for the program into which they were randomized. Cost is estimated as the cost incurred to deliver a program per respondent. Costs are fixed for all respondents who were randomized to receive a given program or its cash equivalent in a given location. Valuation-cost ratio is expressed per 10000 units and capped at 0.001. Standard errors are reported in parenthesis, FWER-adjusted p-values are reported in brackets. * $p < 0.1$, ** $p < 0.05$, *** $p < 0.01$

Table 5: Effect of program allocation and valuation on primary outcome variables for different intervention recipients

	Constant	Program	Valuation	Program X Valuation	N
**PANEL A: AGRICULTURAL EXTENSION RECIPIENTS**					
Monthly Per-Capita Cons (KES)	4236.420***	200.961	−15.899	7.999	961
	(143.050)	(218.738)	(10.377)	(10.821)	
		[0.560]	[0.890]	[0.980]	
Food Security Index	0.044	−0.048	−0.004	0.005	961
	(0.068)	(0.076)	(0.004)	(0.004)	
		[0.560]	[0.950]	[0.960]	
Household Assets (KES)	33478.396***	2344.951	−9.857	86.708	961
	(1545.216)	(2208.797)	(150.491)	(184.437)	
		[0.540]	[0.960]	[0.980]	
Psychological Wellbeing Index	0.013	−0.116*	−0.015***	0.017***	961
	(0.047)	(0.065)	(0.003)	(0.004)	
		[0.260]	[0.050]*	[0.030]**	
Autonomy Index	−0.200***	−0.104	−0.003	0.001	961
	(0.050)	(0.071)	(0.005)	(0.007)	
		[0.410]	[0.950]	[0.980]	
**PANEL B: AGRICULTURAL INPUTS RECIPIENTS**					
	Constant	Program	Valuation	Program X Valuation	N
Monthly Per-Capita Cons (KES)	4949.073***	−680.394**	−10.343	−25.990	959
	(205.112)	(264.652)	(35.052)	(36.742)	
		[0.020]**	[0.990]	[0.810]	
Food Security Index	−0.001	0.047	−0.002	−0.015	959
	(0.041)	(0.062)	(0.008)	(0.010)	
		[0.830]	[0.990]	[0.620]	
Household Assets (KES)	35578.354***	−1962.590	−686.480***	1304.523***	959
	(1516.750)	(2049.133)	(236.732)	(330.673)	
		[0.830]	[0.360]	[0.030]**	
Psychological Wellbeing Index	−0.072	0.035	0.033**	−0.037**	959
	(0.048)	(0.067)	(0.013)	(0.015)	
		[0.830]	[0.040]**	[0.080]*	
Autonomy Index	−0.061	−0.049	−0.014	0.011	959
	(0.054)	(0.076)	(0.013)	(0.015)	
		[0.830]	[0.800]	[0.810]	
**PANEL C: LIVESTOCK RECIPIENTS**					
	Constant	Program	Valuation	Program X Valuation	N
Monthly Per-Capita Cons (KES)	4522.556***	35.422	26.981	−9.502	967
	(198.024)	(279.870)	(39.581)	(51.916)	
		[0.980]	[0.870]	[0.950]	
Food Security Index	0.002	−0.004	−0.011	0.010	967
	(0.044)	(0.065)	(0.011)	(0.014)	
		[0.980]	[0.870]	[0.950]	
Household Assets (KES)	34744.633***	−1361.475	−35.325	−338.610	967
	(1873.191)	(2466.447)	(410.206)	(480.323)	
		[0.940]	[0.930]	[0.950]	
Psychological Wellbeing Index	0.079	−0.120	−0.012	0.014	967
	(0.064)	(0.080)	(0.012)	(0.013)	
		[0.470]	[0.870]	[0.950]	
Autonomy Index	0.339***	−0.265***	−0.015	0.009	967
	(0.054)	(0.078)	(0.013)	(0.014)	
		[0.010]***	[0.840]	[0.950]	

NOTES: Outcome variables are listed on the left. Program is a dummy which takes the value 1 if respondent received a program instead of cash. Valuation is the respondent's stated valuation at baseline for the program into which they were randomized. Valuation is expressed per 10000 KES. Standard errors are reported in parenthesis, FWER-adjusted p-values are reported in brackets. * $p < 0.1$, ** $p < 0.05$, *** $p < 0.01$

Table 6: Effect of recipient preferences being respected in allocation (as per median valuation) on primary outcome variables

	Constant	Respondent preferences respected	N
Monthly Per-Capita Cons (KES)	4341.906***	−85.747	2887
	(131.375)	(130.987)	
		[0.890]	
Food Security Index	0.032	−0.028	2887
	(0.040)	(0.035)	
		[0.890]	
Household Assets (KES)	35241.726***	−821.294	2887
	(1273.707)	(1185.293)	
		[0.890]	
Psychological Wellbeing Index	−0.072*	0.013	2887
	(0.037)	(0.037)	
		[0.890]	
Autonomy Index	−0.231***	−0.055	2887
	(0.037)	(0.038)	
		[0.550]	

NOTES: Outcome variables are listed on the left. Regression includes intervention fixed effects with agricultural extension as omitted category. Respondent preferences respected is an indicator that takes the value 1 if either the respondent values the program less than the median respondent and receives cash, or the respondent values the program more than the median respondent and receives the program. Standard errors are reported in parenthesis, FWER-adjusted p-values are reported in brackets. * $p < 0.1$, ** $p < 0.05$, *** $p < 0.01$

Table 7: Effect of recipient preferences being respected in allocation (as per median valuation) on primary outcome variables for different intervention recipients

	Constant	Respondent preferences respected	N
**PANEL A: AGRICULTURAL EXTENSION RECIPIENTS**			
Monthly Per-Capita Cons (KES)	4361.622***	−121.162	961
	(167.782)	(213.043)	
		[0.820]	
Food Security Index	0.066	−0.089	961
	(0.052)	(0.071)	
		[0.750]	
Household Assets (KES)	35844.632***	−1904.272	961
	(1658.854)	(2150.308)	
		[0.750]	
Psychological Wellbeing Index	−0.077*	0.023	961
	(0.046)	(0.063)	
		[0.820]	
Autonomy Index	−0.217***	−0.081	961
	(0.044)	(0.066)	
		[0.750]	
**PANEL B: AGRICULTURAL INPUTS RECIPIENTS**			
	Constant	Respondent preferences respected	N
Monthly Per-Capita Cons (KES)	4513.273***	82.305	959
	(165.028)	(238.608)	
		[0.830]	
Food Security Index	−0.044	0.079	959
	(0.037)	(0.055)	
		[0.590]	
Household Assets (KES)	32840.937***	2920.433	959
	(1473.770)	(1944.176)	
		[0.590]	
Psychological Wellbeing Index	−0.059	0.053	959
	(0.051)	(0.064)	
		[0.820]	
Autonomy Index	−0.075	−0.043	959
	(0.053)	(0.069)	
		[0.830]	
**PANEL C: LIVESTOCK RECIPIENTS**			
	Constant	Respondent preferences respected	N
Monthly Per-Capita Cons (KES)	4706.012***	−208.967	967
	(169.593)	(228.295)	
		[0.750]	
Food Security Index	0.019	−0.072	967
	(0.040)	(0.055)	
		[0.570]	
Household Assets (KES)	35182.322***	−3386.545*	967
	(1457.988)	(2051.526)	
		[0.420]	
Psychological Wellbeing Index	0.022	−0.034	967
	(0.044)	(0.064)	
		[0.880]	
Autonomy Index	0.195***	−0.036	967
	(0.043)	(0.064)	
		[0.880]	

NOTES: Outcome variables are listed on the left. Respondent preferences respected is an indicator that takes the value 1 if either the respondent values the program less than the median respondent and receives cash, or the respondent values the program more than the median respondent and receives the program. Standard errors are reported in parenthesis, FWER-adjusted p-values are reported in brackets. * $p < 0.1$, ** $p < 0.05$, *** $p < 0.01$

Table 8: Effect of recipient preferences being respected in allocation (as per cost) on primary outcome variables

	Constant	Respondent preferences respected	N
Monthly Per-Capita Cons (KES)	4362.703***	–140.129	2887
	(117.575)	(131.417)	
		[0.540]	
Food Security Index	0.037	–0.041	2887
	(0.044)	(0.035)	
		[0.540]	
Household Assets (KES)	34991.659***	–423.570	2887
	(1188.447)	(1174.823)	
		[0.670]	
Psychological Wellbeing Index	–0.032	–0.067*	2887
	(0.036)	(0.036)	
		[0.230]	
Autonomy Index	–0.207***	–0.111***	2887
	(0.038)	(0.038)	
		[0.000]***	

NOTES: Outcome variables are listed on the left. Regression includes intervention fixed effects with agricultural extension as omitted category. Respondent preferences respected is an indicator that takes the value 1 if either the respondent values the program less than the cost and receives cash, or the respondent values the program more than the cost and receives the program. Standard errors are reported in parenthesis, FWER-adjusted p-values are reported in brackets. * $p < 0.1$, ** $p < 0.05$, *** $p < 0.01$

Table 9: Effect of recipient preferences being respected in allocation (as per cost) on primary outcome variables for different intervention recipients

	Constant	Respondent preferences respected	N
PANEL A: AGRICULTURAL EXTENSION RECIPIENTS			
Monthly Per-Capita Cons (KES)	4176.932***	239.714	961
	(133.132)	(209.271)	
		[0.690]	
Food Security Index	0.039	−0.046	961
	(0.061)	(0.069)	
		[0.780]	
Household Assets (KES)	32797.557***	4062.669*	961
	(1438.638)	(2117.345)	
		[0.300]	
Psychological Wellbeing Index	−0.050	−0.030	961
	(0.044)	(0.063)	
		[0.780]	
Autonomy Index	−0.199***	−0.128*	961
	(0.045)	(0.068)	
		[0.300]	
PANEL B: AGRICULTURAL INPUTS RECIPIENTS	Constant	Respondent preferences respected	N
Monthly Per-Capita Cons (KES)	4756.654***	−385.647	959
	(170.664)	(244.139)	
		[0.400]	
Food Security Index	−0.010	0.026	959
	(0.037)	(0.057)	
		[0.780]	
Household Assets (KES)	35321.287***	−1487.292	959
	(1394.505)	(1926.050)	
		[0.780]	
Psychological Wellbeing Index	−0.005	−0.045	959
	(0.044)	(0.062)	
		[0.780]	
Autonomy Index	−0.069	−0.063	959
	(0.047)	(0.068)	
		[0.750]	
PANEL C: LIVESTOCK RECIPIENTS	Constant	Respondent preferences respected	N
Monthly Per-Capita Cons (KES)	4736.443***	−274.134	967
	(168.051)	(227.914)	
		[0.210]	
Food Security Index	0.034	−0.103*	967
	(0.039)	(0.055)	
		[0.180]	
Household Assets (KES)	35372.593***	−3824.726*	967
	(1446.928)	(2050.640)	
		[0.180]	
Psychological Wellbeing Index	0.066	−0.124*	967
	(0.045)	(0.064)	
		[0.180]	
Autonomy Index	0.248***	−0.143**	967
	(0.042)	(0.064)	
		[0.110]	

NOTES: Outcome variables are listed on the left. Respondent preferences respected is an indicator that takes the value 1 if either the respondent values the program less than the median respondent and receives cash, or the respondent values the program more than the median respondent and receives the program. Standard errors are reported in parenthesis, FWER-adjusted p-values are reported in brackets. * $p < 0.1$, ** $p < 0.05$, *** $p < 0.01$

Table 10: Effect of cash allocation on primary outcome variables

	Constant	Cash	N
Monthly Per-Capita Cons (KES)	4208.517***	169.366	2887
	(128.553)	(131.556)	
		[0.470]	
Food Security Index	0.018	–0.003	2887
	(0.033)	(0.035)	
		[1.000]	
Household Assets (KES)	34946.850***	–321.023	2887
	(1235.305)	(1174.576)	
		[1.000]	
Psychological Wellbeing Index	–0.094***	0.059	2887
	(0.036)	(0.036)	
		[0.310]	
Autonomy Index	–0.325***	0.125***	2887
	(0.040)	(0.038)	
		[0.010]***	

NOTES: Outcome variables are listed on the left. Regression includes intervention fixed effects with agricultural extension as omitted category. Cash is a dummy which takes the value 1 if respondent received a cash transfer instead of a program. Standard errors are reported in parenthesis, FWER-adjusted p-values are reported in brackets. * $p < 0.1$, ** $p < 0.05$, *** $p < 0.01$

Table 11: Effect of cash allocation on primary outcome variables for different intervention recipients

	PANEL A: AGRICULTURAL EXTENSION RECIPIENTS		
	Constant	Cash	N
Monthly Per-Capita Cons (KES)	4403.589***	−216.363	961
	(160.082)	(208.879)	
		[0.680]	
Food Security Index	0.000	0.032	961
	(0.033)	(0.070)	
		[0.680]	
Household Assets (KES)	36152.059***	−2704.162	961
	(1556.959)	(2118.375)	
		[0.570]	
Psychological Wellbeing Index	−0.096**	0.061	961
	(0.044)	(0.063)	
		[0.680]	
Autonomy Index	−0.314***	0.103	961
	(0.050)	(0.068)	
		[0.510]	
	PANEL B: AGRICULTURAL INPUTS RECIPIENTS		
	Constant	Cash	N
Monthly Per-Capita Cons (KES)	4196.565***	733.204***	959
	(159.633)	(243.474)	
		[0.020]**	
Food Security Index	0.011	−0.016	959
	(0.044)	(0.057)	
		[0.950]	
Household Assets (KES)	34842.432***	−545.246	959
	(1336.857)	(1925.988)	
		[0.950]	
Psychological Wellbeing Index	−0.045	0.034	959
	(0.043)	(0.062)	
		[0.950]	
Autonomy Index	−0.115**	0.029	959
	(0.049)	(0.068)	
		[0.950]	
	PANEL C: LIVESTOCK RECIPIENTS		
	Constant	Cash	N
Monthly Per-Capita Cons (KES)	4606.174***	−8.220	967
	(170.791)	(228.491)	
		[0.990]	
Food Security Index	−0.004	−0.024	967
	(0.044)	(0.055)	
		[0.920]	
Household Assets (KES)	32352.067***	2293.847	967
	(1385.686)	(2053.416)	
		[0.680]	
Psychological Wellbeing Index	−0.035	0.081	967
	(0.042)	(0.064)	
		[0.570]	
Autonomy Index	0.058	0.240***	967
	(0.049)	(0.064)	
		[0.000]***	

NOTES: Outcome variables are listed on the left. Cash is a dummy which takes the value 1 if respondent received a cash transfer instead of a program. Standard errors are reported in parenthesis, FWER-adjusted p-values are reported in brackets. * $p < 0.1$, ** $p < 0.05$, *** $p < 0.01$

Table 12: Effect of type of program or cash allocation on primary outcome variables

	(1) Constant	(2) Extension	(3) Cash - Inputs	(4) Inputs	(5) Cash - Livestock	(6) Livestock	(7) Test (p-value): Inputs=Cash	(8) Test (p-value): Livestock=Cash	(9) N
Monthly Per-Capita Cons (KES)	4402.707***	216.363	665.457***	−69.506	195.248	203.468	0.00	0.97	2887
	(285.488)	(208.915)	(234.569)	(240.770)	(323.342)	(332.691)			
		[0.680]	[0.040]**	[0.980]	[0.860]	[0.950]			
Food Security Index	−0.020	−0.032	−0.019	−0.003	−0.009	0.015	0.78	0.66	2887
	(0.092)	(0.070)	(0.073)	(0.072)	(0.098)	(0.102)			
		[0.680]	[0.990]	[0.980]	[0.950]	[0.970]			
Household Assets (KES)	30424.750***	2704.162	1930.791	2500.717	4221.164	1927.317	0.77	0.26	2887
	(2377.289)	(2118.740)	(2168.888)	(2138.282)	(2819.346)	(2751.784)			
		[0.510]	[0.860]	[0.700]	[0.480]	[0.950]			
Psychological Wellbeing Index	−0.015	−0.061	0.017	−0.018	0.060	−0.021	0.58	0.21	2887
	(0.080)	(0.063)	(0.066)	(0.065)	(0.093)	(0.090)			
		[0.680]	[0.990]	[0.980]	[0.860]	[0.970]			
Autonomy Index	0.126	−0.103	0.004	−0.028	0.171*	−0.068	0.63	0.00	2887
	(0.082)	(0.068)	(0.071)	(0.072)	(0.092)	(0.096)			
		[0.440]	[0.990]	[0.980]	[0.300]	[0.950]			

NOTES: Outcome variables are listed on the left. Regression includes location fixed effects with Kilungu as omitted category. Receiving a cash transfer equivalent to extension is the omitted category. Column (2) Extension refers to respondents randomized to receive the agricutural extension program. Column (3) Cash - Inputs refers to respondents randomized to receive a cash transfer equivalent to inputs. Column (4) Inputs refers to respondents randomized to receive the agricultural inputs program. Column (5) Cash - Livestock refers to respondents randomized to receive a cash transfer equivalent to the livestock package. Column (6) Livestock refers to respondents randomized to receive the livestock package. Column (7) displays the p-value from test for equality of coefficients of inputs and its cash equivalent and column (8) for livestock and its cash equivalent. Standard errors are reported in parenthesis, FWER-adjusted p-values are reported in brackets. * $p < 0.1$, ** $p < 0.05$, *** $p < 0.01$

Table 13: Multivariate regression of primary outcome variables on indicator for recipient preferences being respected in allocation (as per median valuation) and age

	Constant	Resp pref respected	Age	Resp pref respected X Age	N
Monthly Per-Capita Cons (KES)	3853.878***	−845.753**	11.208	16.924*	2887
	(344.210)	(415.423)	(7.056)	(9.093)	
		[0.150]	[0.300]	[0.150]	
Food Security Index	0.124*	−0.019	−0.002	−0.000	2887
	(0.067)	(0.092)	(0.001)	(0.002)	
		[0.900]	[0.460]	[0.930]	
Household Assets (KES)	29721.455***	−2235.162	127.412**	29.053	2887
	(2777.756)	(3404.487)	(58.910)	(75.815)	
		[0.800]	[0.140]	[0.900]	
Psychological Wellbeing Index	0.009	0.102	−0.002	−0.002	2887
	(0.088)	(0.113)	(0.002)	(0.002)	
		[0.800]	[0.460]	[0.770]	
Autonomy Index	−0.121	−0.301***	−0.003	0.006**	2887
	(0.083)	(0.111)	(0.002)	(0.002)	
		[0.080]*	[0.460]	[0.130]	

NOTES: Outcome variables are listed on the left. Respondent preferences respected is an indicator that takes the value 1 if either the respondent values the program less than the median respondent and receives cash, or the respondent values the program more than the median respondent and receives the program. Age of respondent is as reported at baseline. Standard errors are reported in parenthesis, FWER-adjusted p-values are reported in brackets. * $p < 0.1$, ** $p < 0.05$, *** $p < 0.01$

Table 14: Multivariate regression of primary outcome variables on indicator for recipient preferences being respected in allocation (as per cost) and age

	Constant	Resp pref respected	Age	Resp pref respected X Age	N
Monthly Per-Capita Cons (KES)	3464.572***	−164.402	20.567***	0.384	2887
	(300.841)	(406.411)	(6.548)	(8.932)	
		[1.000]	[0.000]***	[0.980]	
Food Security Index	0.116	−0.004	−0.002	−0.001	2887
	(0.082)	(0.095)	(0.001)	(0.002)	
		[1.000]	[0.430]	[0.960]	
Household Assets (KES)	29677.700***	−2352.401	121.911**	42.879	2887
	(2607.235)	(3343.601)	(55.607)	(74.215)	
		[1.000]	[0.060]*	[0.950]	
Psychological Wellbeing Index	0.075	−0.024	−0.002	−0.001	2887
	(0.085)	(0.112)	(0.002)	(0.002)	
		[1.000]	[0.430]	[0.960]	
Autonomy Index	−0.304***	0.030	0.002	−0.003	2887
	(0.081)	(0.111)	(0.002)	(0.002)	
		[1.000]	[0.430]	[0.700]	

NOTES: Outcome variables are listed on the left. Respondent preferences respected is an indicator that takes the value 1 if either the respondent values the program less than the cost and receives cash, or the respondent values the program more than the cost and receives the program. Age of respondent is as reported at baseline. Standard errors are reported in parenthesis, FWER-adjusted p-values are reported in brackets. * $p < 0.1$, ** $p < 0.05$, *** $p < 0.01$

Table 15: Multivariate regression of primary outcome variables on indicator for recipient preferences being respected in allocation (as per median valuation) and gender

	Constant	Resp pref respected	Female	Resp pref respected X Female	N
Monthly Per-Capita Cons (KES)	5121.252***	−183.543	−1327.956***	201.531	2887
	(216.476)	(257.956)	(216.354)	(292.213)	
		[0.750]	[0.000]***	[0.820]	
Food Security Index	0.040	−0.030	−0.013	0.003	2887
	(0.044)	(0.049)	(0.051)	(0.069)	
		[0.830]	[0.920]	[0.960]	
Household Assets (KES)	40184.218***	−3259.846	−8339.193***	4179.458*	2887
	(1693.392)	(2016.916)	(1834.606)	(2475.336)	
		[0.390]	[0.000]***	[0.300]	
Psychological Wellbeing Index	0.002	−0.064	−0.123**	0.128*	2887
	(0.049)	(0.059)	(0.054)	(0.075)	
		[0.710]	[0.080]*	[0.300]	
Autonomy Index	−0.244***	−0.019	0.020	−0.059	2887
	(0.049)	(0.062)	(0.054)	(0.079)	
		[0.830]	[0.920]	[0.820]	

NOTES: Outcome variables are listed on the left. Respondent preferences respected is an indicator that takes the value 1 if either the respondent values the program less than the median respondent and receives cash, or the respondent values the program more than the median respondent and receives the program. Female is an indicator for respondent being female. Standard errors are reported in parenthesis, FWER-adjusted p-values are reported in brackets. * $p < 0.1$, ** $p < 0.05$, *** $p < 0.01$

Table 16: Multivariate regression of primary outcome variables on indicator for recipient preferences being respected in allocation (as per cost) and gender

	Constant	Resp pref respected	Female	Resp pref respected X Female	N
Monthly Per-Capita Cons (KES)	5026.979***	−9.658	−1132.917***	−172.453	2887
	(179.153)	(256.757)	(199.770)	(291.646)	
		[0.980]	[0.000]***	[0.870]	
Food Security Index	0.057	−0.069	−0.034	0.045	2887
	(0.041)	(0.049)	(0.053)	(0.070)	
		[0.480]	[0.570]	[0.870]	
Household Assets (KES)	39654.818***	−2646.250	−7941.996***	3802.481	2887
	(1615.765)	(2014.359)	(1751.305)	(2467.286)	
		[0.430]	[0.000]***	[0.420]	
Psychological Wellbeing Index	0.001	−0.071	−0.056	0.008	2887
	(0.047)	(0.058)	(0.052)	(0.074)	
		[0.480]	[0.570]	[0.910]	
Autonomy Index	−0.179***	−0.158**	−0.048	0.075	2887
	(0.047)	(0.063)	(0.052)	(0.079)	
		[0.010]***	[0.570]	[0.850]	

NOTES: Outcome variables are listed on the left. Respondent preferences respected is an indicator that takes the value 1 if either the respondent values the program less than the cost and receives cash, or the respondent values the program more than the cost and receives the program. Female is an indicator for respondent being female. Standard errors are reported in parenthesis, FWER-adjusted p-values are reported in brackets. * $p < 0.1$, ** $p < 0.05$, *** $p < 0.01$

Table 17: Multivariate regression of primary outcome variables on indicator for recipient preferences being respected in allocation (as per median valuation) and wealth

	Constant	Resp pref respected	Household wealth	Resp pref respected X Household wealth	N
Monthly Per-Capita Cons (KES)	4349.873***	−79.779	−0.000	−0.000	2887
	(132.063)	(135.164)	(0.000)	(0.000)	
		[0.870]	[0.890]	[0.870]	
Food Security Index	0.036	−0.025	−0.000	−0.000	2887
	(0.040)	(0.036)	(0.000)	(0.000)	
		[0.870]	[0.890]	[0.870]	
Household Assets (KES)	35379.117***	−1777.214	−0.000*	0.001**	2887
	(1279.169)	(1218.150)	(0.000)	(0.000)	
		[0.410]	[0.500]	[0.000]***	
Psychological Wellbeing Index	−0.071*	0.020	−0.000	−0.000	2887
	(0.037)	(0.038)	(0.000)	(0.000)	
		[0.870]	[0.890]	[0.840]	
Autonomy Index	−0.221***	−0.071*	−0.000***	0.000	2887
	(0.038)	(0.040)	(0.000)	(0.000)	
		[0.260]	[0.150]	[0.440]	

NOTES: Outcome variables are listed on the left. Respondent preferences respected is an indicator that takes the value 1 if either the respondent values the program less than the median respondent and receives cash, or the respondent values the program more than the median respondent and receives the program. Wealth is value of all household assets (in KES) reported at baseline. Standard errors are reported in parenthesis, FWER-adjusted p-values are reported in brackets. * $p < 0.1$, ** $p < 0.05$, *** $p < 0.01$

Table 18: Multivariate regression of primary outcome variables on indicator for recipient preferences being respected in allocation (as per cost) and wealth

	Constant	Resp pref respected	Household wealth	Resp pref respected X Household wealth	N
Monthly Per-Capita Cons (KES)	4370.118***	–118.029	–0.000	–0.000	2887
	(117.960)	(134.428)	(0.000)	(0.000)	
		[0.560]	[0.940]	[0.870]	
Food Security Index	0.040	–0.040	–0.000	–0.000	2887
	(0.045)	(0.036)	(0.000)	(0.000)	
		[0.560]	[0.900]	[0.950]	
Household Assets (KES)	35114.351***	–1384.966	–0.000	0.001**	2887
	(1192.853)	(1204.458)	(0.000)	(0.000)	
		[0.560]	[0.370]	[0.000]***	
Psychological Wellbeing Index	–0.030	–0.065*	–0.000	–0.000	2887
	(0.036)	(0.037)	(0.000)	(0.000)	
		[0.300]	[0.940]	[0.950]	
Autonomy Index	–0.197***	–0.128***	–0.000***	0.000	2887
	(0.038)	(0.040)	(0.000)	(0.000)	
		[0.000]***	[0.060]*	[0.460]	

NOTES: Outcome variables are listed on the left. Respondent preferences respected is an indicator that takes the value 1 if either the respondent values the program less than the cost and receives cash, or the respondent values the program more than the cost and receives the program. Wealth is value of all household assets (in KES) reported at baseline. Standard errors are reported in parenthesis, FWER-adjusted p-values are reported in brackets. * $p < 0.1$, ** $p < 0.05$, *** $p < 0.01$

Table 19: Multivariate regression of primary outcome variables on indicator for recipient preferences being respected in allocation (as per median valuation) and growth mindset

	Constant	Resp pref respected	Growth mindset	Resp pref respected X Growth mindset	N
Monthly Per-Capita Cons (KES)	5056.880***	−738.113	−22.277*	20.289	2887
	(428.406)	(555.429)	(12.074)	(16.248)	
		[0.720]	[0.290]	[0.600]	
Food Security Index	0.090	−0.214*	−0.002	0.006	2887
	(0.101)	(0.125)	(0.003)	(0.004)	
		[0.700]	[0.850]	[0.590]	
Household Assets (KES)	24485.173***	−1512.720	337.470***	21.161	2887
	(3539.750)	(4782.568)	(106.468)	(149.141)	
		[0.990]	[0.030]**	[0.990]	
Psychological Wellbeing Index	−0.215*	−0.063	0.004	0.002	2887
	(0.113)	(0.155)	(0.003)	(0.005)	
		[0.990]	[0.480]	[0.950]	
Autonomy Index	−0.267**	−0.029	0.001	−0.001	2887
	(0.113)	(0.161)	(0.003)	(0.005)	
		[0.990]	[0.850]	[0.990]	

NOTES: Outcome variables are listed on the left. Respondent preferences respected is an indicator that takes the value 1 if either the respondent values the program less than the median respondent and receives cash, or the respondent values the program more than the median respondent and receives the program. A higher value of the mindset index indicates a growth mindset as opposed to a fixed mindset of the respondent. Standard errors are reported in parenthesis, FWER-adjusted p-values are reported in brackets. * $p < 0.1$, ** $p < 0.05$, *** $p < 0.01$

Table 20: Multivariate regression of primary outcome variables on indicator for recipient preferences being respected in allocation (as per cost) and growth mindset

	Constant	Resp pref respected	Growth mindset	Resp pref respected X Growth mindset	N
Monthly Per-Capita Cons (KES)	4826.927***	–344.943	–14.526	6.381	2887
	(399.653)	(552.480)	(11.467)	(16.199)	
		[0.900]	[0.480]	[1.000]	
Food Security Index	–0.017	–0.018	0.002	–0.001	2887
	(0.099)	(0.124)	(0.003)	(0.004)	
		[0.960]	[0.650]	[1.000]	
Household Assets (KES)	25916.915***	–4616.100	285.315***	129.811	2887
	(3374.820)	(4779.312)	(100.790)	(149.293)	
		[0.860]	[0.060]*	[0.890]	
Psychological Wellbeing Index	–0.232**	–0.034	0.006*	–0.001	2887
	(0.110)	(0.155)	(0.003)	(0.005)	
		[0.960]	[0.250]	[1.000]	
Autonomy Index	–0.375***	0.186	0.005	–0.009*	2887
	(0.117)	(0.162)	(0.003)	(0.005)	
		[0.840]	[0.400]	[0.250]	

NOTES: Outcome variables are listed on the left. Respondent preferences respected is an indicator that takes the value 1 if either the respondent values the program less than the cost and receives cash, or the respondent values the program more than the cost and receives the program. A higher value of the mindset index indicates a growth mindset as opposed to a fixed mindset of the respondent. Standard errors are reported in parenthesis, FWER-adjusted p-values are reported in brackets. * $p < 0.1$, ** $p < 0.05$, *** $p < 0.01$

Table 21: Multivariate regression of primary outcome variables on indicator for recipient preferences being respected in allocation (as per median valuation) and grit

	Constant	Resp pref respected	Grit	Resp pref respected X Grit	N
Monthly Per-Capita Cons (KES)	3974.205***	715.664	108.823	–235.693	2887
	(684.987)	(895.673)	(200.849)	(260.801)	
		[0.790]	[0.910]	[0.770]	
Food Security Index	0.062	–0.351	–0.009	0.095	2887
	(0.143)	(0.236)	(0.044)	(0.069)	
		[0.570]	[0.970]	[0.610]	
Household Assets (KES)	24013.932***	2090.179	3320.452*	–877.176	2887
	(5889.447)	(7663.461)	(1705.802)	(2237.885)	
		[0.950]	[0.270]	[0.940]	
Psychological Wellbeing Index	–0.234	–0.270	0.048	0.083	2887
	(0.187)	(0.254)	(0.055)	(0.074)	
		[0.660]	[0.770]	[0.710]	
Autonomy Index	–0.188	0.015	–0.013	–0.020	2887
	(0.180)	(0.265)	(0.052)	(0.078)	
		[0.960]	[0.970]	[0.940]	

NOTES: Outcome variables are listed on the left. Respondent preferences respected is an indicator that takes the value 1 if either the respondent values the program less than the median respondent and receives cash, or the respondent values the program more than the median respondent and receives the program. A higher value of the grit index indicates higher grittiness in respondent's personality. Standard errors are reported in parenthesis, FWER-adjusted p-values are reported in brackets. * $p < 0.1$, ** $p < 0.05$, *** $p < 0.01$

Table 22: Multivariate regression of primary outcome variables on indicator for recipient preferences being respected in allocation (as per cost) and grit

	Constant	Resp pref respected	Grit	Resp pref respected X Grit	N
Monthly Per-Capita Cons (KES)	4801.628***	–906.818	–129.542	225.600	2887
	(632.157)	(881.904)	(180.970)	(255.619)	
		[0.910]	[0.860]	[0.970]	
Food Security Index	–0.072	–0.124	0.032	0.024	2887
	(0.178)	(0.239)	(0.048)	(0.070)	
		[0.990]	[0.860]	[0.980]	
Household Assets (KES)	24861.791***	632.455	2996.941*	–337.441	2887
	(5354.392)	(7589.486)	(1555.512)	(2213.283)	
		[0.990]	[0.220]	[0.980]	
Psychological Wellbeing Index	–0.326*	–0.132	0.087*	0.018	2887
	(0.180)	(0.253)	(0.053)	(0.074)	
		[0.990]	[0.240]	[0.980]	
Autonomy Index	–0.236	0.099	0.009	–0.062	2887
	(0.170)	(0.269)	(0.049)	(0.080)	
		[0.990]	[0.900]	[0.970]	

NOTES: Outcome variables are listed on the left. Respondent preferences respected is an indicator that takes the value 1 if either the respondent values the program less than the cost and receives cash, or the respondent values the program more than the cost and receives the program. A higher value of the grit index indicates higher grittiness in respondent's personality. Standard errors are reported in parenthesis, FWER-adjusted p-values are reported in brackets. * $p < 0.1$, ** $p < 0.05$, *** $p < 0.01$

Table 23: Multivariate regression of primary outcome variables on indicator for cash allocation and age

	Constant	Cash	Age	Cash X Age	N
Monthly Per-Capita Cons (KES)	3448.758***	–130.691	17.317***	6.778	2887
	(305.544)	(407.097)	(6.057)	(8.953)	
		[0.970]	[0.030]**	[0.940]	
Food Security Index	0.150**	–0.073	–0.003**	0.002	2887
	(0.070)	(0.096)	(0.001)	(0.002)	
		[0.920]	[0.200]	[0.940]	
Household Assets (KES)	27638.531***	1811.261	167.295***	–49.249	2887
	(2428.145)	(3359.661)	(50.175)	(74.557)	
		[0.970]	[0.010]***	[0.940]	
Psychological Wellbeing Index	0.003	0.126	–0.002	–0.002	2887
	(0.080)	(0.112)	(0.002)	(0.002)	
		[0.840]	[0.330]	[0.940]	
Autonomy Index	–0.274***	–0.029	–0.001	0.004	2887
	(0.084)	(0.111)	(0.002)	(0.002)	
		[0.970]	[0.480]	[0.610]	

NOTES: Outcome variables are listed on the left. Cash is a dummy which takes the value 1 if respondent received a cash transfer instead of a program. Age of respondent is as reported at baseline. Standard errors are reported in parenthesis, FWER-adjusted p-values are reported in brackets. * $p < 0.1$, ** $p < 0.05$, *** $p < 0.01$

Table 24: Multivariate regression of primary outcome variables on indicator for cash allocation and gender

	Constant	Cash	Female	Cash X Female	N
Monthly Per-Capita Cons (KES)	4869.421***	292.149	−1101.720***	−229.295	2887
	(207.859)	(256.397)	(210.029)	(291.438)	
		[0.490]	[0.000]***	[0.790]	
Food Security Index	0.031	−0.012	−0.020	0.016	2887
	(0.045)	(0.049)	(0.049)	(0.070)	
		[0.870]	[0.780]	[0.920]	
Household Assets (KES)	38897.924***	−930.435	−6515.314***	839.230	2887
	(1678.546)	(2018.945)	(1717.310)	(2471.381)	
		[0.870]	[0.000]***	[0.920]	
Psychological Wellbeing Index	−0.011	−0.041	−0.133***	0.160**	2887
	(0.048)	(0.058)	(0.051)	(0.074)	
		[0.850]	[0.020]**	[0.140]	
Autonomy Index	−0.361***	0.206***	0.056	−0.132*	2887
	(0.057)	(0.062)	(0.060)	(0.079)	
		[0.010]***	[0.580]	[0.270]	

NOTES: Outcome variables are listed on the left. Cash is a dummy which takes the value 1 if respondent received a cash transfer instead of a program. Female is an indicator for respondent being female. Standard errors are reported in parenthesis, FWER-adjusted p-values are reported in brackets. * $p < 0.1$, ** $p < 0.05$, *** $p < 0.01$

Table 25: Multivariate regression of primary outcome variables on indicator for cash allocation and wealth

	Constant	Cash	Household wealth	Cash X Household wealth	N
Monthly Per-Capita Cons (KES)	4214.083***	171.438	−0.000	−0.000	2887
	(130.315)	(133.140)	(0.000)	(0.000)	
		[0.520]	[1.000]	[1.000]	
Food Security Index	0.021	−0.003	−0.000	0.000	2887
	(0.033)	(0.036)	(0.000)	(0.000)	
		[0.980]	[0.980]	[1.000]	
Household Assets (KES)	34556.371***	290.968	0.001***	−0.001***	2887
	(1239.591)	(1181.152)	(0.000)	(0.000)	
		[0.980]	[0.120]	[0.020]**	
Psychological Wellbeing Index	−0.094**	0.060	−0.000	−0.000	2887
	(0.037)	(0.037)	(0.000)	(0.000)	
		[0.330]	[1.000]	[1.000]	
Autonomy Index	−0.315***	0.123***	−0.000	0.000	2887
	(0.040)	(0.039)	(0.000)	(0.000)	
		[0.000]***	[0.620]	[1.000]	

NOTES: Outcome variables are listed on the left. Cash is a dummy which takes the value 1 if respondent received a cash transfer instead of a program. Wealth is value of all household assets (in KES) reported at baseline. Standard errors are reported in parenthesis, FWER-adjusted p-values are reported in brackets. * $p < 0.1$, ** $p < 0.05$, *** $p < 0.01$

Table 26: Multivariate regression of primary outcome variables on indicator for cash allocation and growth mindset

	Constant	Cash	Growth mindset	Cash X Growth mindset	N
Monthly Per-Capita Cons (KES)	4397.198***	529.569	−6.004	−11.103	2887
	(391.600)	(553.799)	(11.191)	(16.230)	
		[0.770]	[0.950]	[0.580]	
Food Security Index	0.044	−0.137	−0.001	0.004	2887
	(0.091)	(0.124)	(0.003)	(0.004)	
		[0.770]	[0.980]	[0.580]	
Household Assets (KES)	20021.901***	7160.950	468.300***	−235.318	2887
	(3443.819)	(4779.099)	(107.617)	(149.250)	
		[0.580]	[0.000]***	[0.450]	
Psychological Wellbeing Index	−0.115	−0.261*	0.001	0.010**	2887
	(0.109)	(0.154)	(0.003)	(0.005)	
		[0.500]	[0.980]	[0.190]	
Autonomy Index	−0.234*	−0.088	−0.003	0.007	2887
	(0.119)	(0.163)	(0.004)	(0.005)	
		[0.770]	[0.880]	[0.500]	

NOTES: Outcome variables are listed on the left. Cash is a dummy which takes the value 1 if respondent received a cash transfer instead of a program. A higher value of the mindset index indicates a growth mindset as opposed to a fixed mindset of the respondent. Standard errors are reported in parenthesis, FWER-adjusted p-values are reported in brackets. * $p < 0.1$, ** $p < 0.05$, *** $p < 0.01$

Table 27: Multivariate regression of primary outcome variables on indicator for cash allocation and grit

	Constant	Cash	Grit	Cash X Grit	N
Monthly Per-Capita Cons (KES)	3888.499***	946.006	93.697	–228.605	2887
	(586.401)	(882.574)	(168.088)	(256.005)	
		[0.820]	[0.840]	[0.860]	
Food Security Index	–0.063	–0.133	0.024	0.039	2887
	(0.171)	(0.239)	(0.050)	(0.070)	
		[0.980]	[0.840]	[0.960]	
Household Assets (KES)	23825.630***	2937.427	3256.964**	–927.062	2887
	(5302.529)	(7602.962)	(1518.530)	(2218.057)	
		[0.980]	[0.170]	[0.960]	
Psychological Wellbeing Index	–0.365**	–0.056	0.079	0.035	2887
	(0.173)	(0.253)	(0.050)	(0.074)	
		[0.980]	[0.360]	[0.960]	
Autonomy Index	–0.159	–0.072	–0.049	0.058	2887
	(0.207)	(0.267)	(0.061)	(0.079)	
		[0.980]	[0.720]	[0.870]	

NOTES: Outcome variables are listed on the left. Cash is a dummy which takes the value 1 if respondent received a cash transfer instead of a program. A higher value of the grit index indicates higher grittiness in respondent's personality. Standard errors are reported in parenthesis, FWER-adjusted p-values are reported in brackets. * $p < 0.1$, ** $p < 0.05$, *** $p < 0.01$

Table 28: Effect of program allocation and valuation-cost ratio on secondary outcome variables

	Constant	Program	Valuation-cost ratio	Program X Valuation-cost ratio	N
Monthly Household Income (KES)	11128.421**	−9546.388	−394031.169	468968.534	2887
	(4677.062)	(8893.675)	(404282.571)	(485070.527)	
		[0.550]	[1.000]	[1.000]	
Paid Activities (Hours/Week)	32.288***	−0.480	270.815	−202.179	2887
	(1.229)	(1.158)	(234.114)	(246.865)	
		[0.680]	[0.680]	[0.990]	
Education Index	−0.106**	−0.116***	−19.068**	28.862***	2307
	(0.043)	(0.043)	(7.792)	(9.190)	
		[0.020]**	[0.060]*	[0.020]**	

NOTES: Outcome variables are listed on the left. Regression includes intervention fixed effects with agricultural extension as omitted category. Program is a dummy which takes the value 1 if respondent received a program instead of cash. Valuation is the respondent's stated valuation at baseline for the program into which they were randomized. Cost is estimated as the cost incurred to deliver a program per respondent. Costs are fixed for all respondents who were randomized to receive a given program or its cash equivalent in a given location. Valuation-cost ratio is expressed per 10000 units. Standard errors are reported in parenthesis, FWER-adjusted p-values are reported in brackets. * $p < 0.1$, ** $p < 0.05$, *** $p < 0.01$

Table 29: Effect of program allocation and winsorized valuation-cost ratio on secondary outcome variables

	Constant	Program	Valuation-cost ratio (trimmed)	Program X Valuation-cost ratio (trimmed)	N
Monthly Household Income (KES)	13283.932**	–12197.883	–3665826.933	5401119.745	2887
	(6528.294)	(11502.215)	(3371909.673)	(5335392.366)	
		[0.380]	[1.000]	[1.000]	
Paid Activities (Hours/Week)	31.812***	0.385	1250.961	–1796.006	2887
	(1.578)	(1.421)	(1649.169)	(1761.505)	
		[0.780]	[0.500]	[0.570]	
Education Index	–0.076	–0.109**	–52.270	36.693	2307
	(0.055)	(0.051)	(39.705)	(55.153)	
		[0.050]*	[0.420]	[1.000]	

NOTES: Outcome variables are listed on the left. Regression includes intervention fixed effects with agricultural extension as omitted category. Program is a dummy which takes the value 1 if respondent received a program instead of cash. Valuation is the respondent's stated valuation at baseline for the program into which they were randomized. Cost is estimated as the cost incurred to deliver a program per respondent. Costs are fixed for all respondents who were randomized to receive a given program or its cash equivalent in a given location. Valuation-cost ratio is expressed per 10000 units and winsorized at the 95th percentile. Standard errors are reported in parenthesis, FWER-adjusted p-values are reported in brackets. * $p < 0.1$, ** $p < 0.05$, *** $p < 0.01$

Table 30: Effect of program allocation and log of valuation-cost ratio on secondary outcome variables

	Constant	Program	ln(Valuation-cost ratio)	Program X ln(Valuation-cost ratio)	N
Monthly Household Income (KES)	12286.489**	−12167.793	−1517.041	2928.046	2887
	(5389.700)	(11362.532)	(1461.194)	(2859.998)	
		[0.480]	[1.000]	[1.000]	
Paid Activities (Hours/Week)	31.428***	0.190	0.819	−0.785	2887
	(1.690)	(1.484)	(0.892)	(0.985)	
		[0.900]	[0.600]	[0.840]	
Education Index	−0.068	−0.099*	−0.031	0.010	2307
	(0.060)	(0.056)	(0.026)	(0.036)	
		[0.120]	[0.600]	[1.000]	

NOTES: Outcome variables are listed on the left. Regression includes intervention fixed effects with agricultural extension as omitted category. Program is a dummy which takes the value 1 if respondent received a program instead of cash. Valuation is the respondent's stated valuation at baseline for the program into which they were randomized. Cost is estimated as the cost incurred to deliver a program per respondent. Costs are fixed for all respondents who were randomized to receive a given program or its cash equivalent in a given location. Valuation-cost ratio is expressed per 10000 units. Standard errors are reported in parenthesis, FWER-adjusted p-values are reported in brackets. * $p < 0.1$, ** $p < 0.05$, *** $p < 0.01$

Table 31: Effect of program allocation and capped valuation-cost ratio on secondary outcome variables

	Constant	Program	Valuation-cost ratio (capped)	Program X Valuation-cost ratio (capped)	N
Monthly Household Income (KES)	17448.979*	–16123.121	–13954199.686	18152220.414	2887
	(9969.063)	(15089.147)	(12221281.521)	(17233790.240)	
		[0.440]	[1.000]	[0.970]	
Paid Activities (Hours/Week)	30.949***	1.264	3686.092	–4932.358	2887
	(1.952)	(1.745)	(3316.600)	(3724.061)	
		[0.550]	[0.370]	[0.270]	
Education Index	–0.056	–0.068	–94.351	–53.869	2307
	(0.071)	(0.065)	(105.082)	(135.533)	
		[0.550]	[0.650]	[0.970]	

NOTES: Outcome variables are listed on the left. Regression includes intervention fixed effects with agricultural extension as omitted category. Program is a dummy which takes the value 1 if respondent received a program instead of cash. Valuation is the respondent's stated valuation at baseline for the program into which they were randomized. Cost is estimated as the cost incurred to deliver a program per respondent. Costs are fixed for all respondents who were randomized to receive a given program or its cash equivalent in a given location. Valuation-cost ratio is expressed per 10000 units and capped at 0.001. Standard errors are reported in parenthesis, FWER-adjusted p-values are reported in brackets. * $p < 0.1$, ** $p < 0.05$, *** $p < 0.01$

Table 32: Effect of program allocation and valuation on secondary outcome variables for different intervention recipients

	Constant	Program	Valuation	Program X Valuation	N
PANEL A: AGRICULTURAL EXTENSION RECIPIENTS					
Monthly Household Income (KES)	5697.812***	835.573	20.026	−33.658	961
	(497.346)	(895.380)	(43.122)	(47.168)	
		[0.550]	[0.830]	[0.880]	
Paid Activities (Hours/Week)	33.032***	−1.953	0.142	−0.081	961
	(1.602)	(2.024)	(0.151)	(0.160)	
		[0.550]	[0.800]	[0.880]	
Education Index	−0.083	−0.166**	−0.012**	0.019***	788
	(0.051)	(0.075)	(0.005)	(0.006)	
		[0.030]**	[0.250]	[0.040]**	
PANEL B: AGRICULTURAL INPUTS RECIPIENTS					
	Constant	Program	Valuation	Program X Valuation	N
Monthly Household Income (KES)	12175.663**	−4682.158	−286.217	205.637	959
	(5327.952)	(5402.023)	(527.966)	(531.249)	
		[0.690]	[1.000]	[1.000]	
Paid Activities (Hours/Week)	34.025***	0.101	0.199	−0.519	959
	(1.831)	(2.373)	(0.481)	(0.502)	
		[0.980]	[1.000]	[0.860]	
Education Index	0.041	−0.147*	−0.026	0.017	769
	(0.063)	(0.085)	(0.017)	(0.019)	
		[0.200]	[0.220]	[0.860]	
PANEL C: LIVESTOCK RECIPIENTS					
	Constant	Program	Valuation	Program X Valuation	N
Monthly Household Income (KES)	35662.995	−28787.824	−1833.682	1811.918	967
	(30540.962)	(30546.627)	(2029.192)	(2030.371)	
		[0.990]	[1.000]	[1.000]	
Paid Activities (Hours/Week)	28.646***	1.944	0.225	−0.310	967
	(1.438)	(2.027)	(0.310)	(0.353)	
		[0.990]	[1.000]	[1.000]	
Education Index	0.148**	−0.022	−0.004	−0.010	750
	(0.067)	(0.091)	(0.017)	(0.020)	
		[0.990]	[1.000]	[1.000]	

NOTES: Outcome variables are listed on the left. Program is a dummy which takes the value 1 if respondent received a program instead of cash. Valuation is the respondent's stated valuation at baseline for the program into which they were randomized. Valuation is expressed per 10000 KES. Standard errors are reported in parenthesis, FWER-adjusted p-values are reported in brackets. * $p < 0.1$, ** $p < 0.05$, *** $p < 0.01$

Table 33: Effect of recipient preferences being respected in allocation (as per median valuation) on secondary outcome variables

	Constant	Respondent preferences respected	N
Monthly Household Income (KES)	10607.001**	–8071.807	2887
	(4769.814)	(8521.764)	
		[0.810]	
Paid Activities (Hours/Week)	32.734***	–0.566	2887
	(1.270)	(1.159)	
		[0.830]	
Education Index	–0.158***	–0.022	2307
	(0.043)	(0.043)	
		[0.830]	

NOTES: Outcome variables are listed on the left. Regression includes intervention fixed effects with agricultural extension as omitted category. Respondent preferences respected is an indicator that takes the value 1 if either the respondent values the program less than the median respondent and receives cash, or the respondent values the program more than the median respondent and receives the program. Standard errors are reported in parenthesis, FWER-adjusted p-values are reported in brackets. * $p < 0.1$, ** $p < 0.05$, *** $p < 0.01$

Table 34: Effect of recipient preferences being respected in allocation (as per median valuation) on secondary outcome variables for different intervention recipients

	PANEL A: AGRICULTURAL EXTENSION RECIPIENTS		
	Constant	Respondent preferences respected	N
Monthly Household Income (KES)	6456.420***	−616.278	961
	(753.278)	(885.678)	
		[0.830]	
Paid Activities (Hours/Week)	32.988***	−1.022	961
	(1.701)	(2.090)	
		[0.830]	
Education Index	−0.149***	−0.037	788
	(0.052)	(0.073)	
		[0.830]	
	PANEL B: AGRICULTURAL INPUTS RECIPIENTS		
	Constant	Respondent preferences respected	N
Monthly Household Income (KES)	8118.795***	2299.577	959
	(1094.842)	(3888.934)	
		[0.980]	
Paid Activities (Hours/Week)	33.350***	0.998	959
	(1.622)	(2.237)	
		[0.980]	
Education Index	−0.043	−0.039	769
	(0.061)	(0.080)	
		[0.980]	
	PANEL C: LIVESTOCK RECIPIENTS		
	Constant	Respondent preferences respected	N
Monthly Household Income (KES)	31298.264	−25398.570	967
	(24734.625)	(24737.707)	
		[0.600]	
Paid Activities (Hours/Week)	30.649***	−1.680	967
	(1.199)	(1.681)	
		[0.600]	
Education Index	0.099*	0.024	750
	(0.051)	(0.070)	
		[0.670]	

NOTES: Outcome variables are listed on the left. Respondent preferences respected is an indicator that takes the value 1 if either the respondent values the program less than the median respondent and receives cash, or the respondent values the program more than the median respondent and receives the program. Standard errors are reported in parenthesis, FWER-adjusted p-values are reported in brackets. * $p < 0.1$, ** $p < 0.05$, *** $p < 0.01$

Table 35: Effect of recipient preferences being respected in allocation (as per cost) on secondary outcome variables

	Constant	Respondent preferences respected	N
Monthly Household Income (KES)	9361.350**	–6641.162	2887
	(4081.334)	(8308.534)	
		[0.900]	
Paid Activities (Hours/Week)	32.838***	–0.857	2887
	(1.236)	(1.151)	
		[0.900]	
Education Index	–0.145***	–0.050	2307
	(0.041)	(0.043)	
		[0.540]	

NOTES: Outcome variables are listed on the left. Regression includes intervention fixed effects with agricultural extension as omitted category. Respondent preferences respected is an indicator that takes the value 1 if either the respondent values the program less than the cost and receives cash, or the respondent values the program more than the cost and receives the program. Standard errors are reported in parenthesis, FWER-adjusted p-values are reported in brackets. * $p < 0.1$, ** $p < 0.05$, *** $p < 0.01$

Table 36: Effect of recipient preferences being respected in allocation (as per cost) on secondary outcome variables for different intervention recipients

	PANEL A: AGRICULTURAL EXTENSION RECIPIENTS		
	Constant	Respondent preferences respected	N
Monthly Household Income (KES)	5637.745***	972.420	961
	(456.956)	(853.013)	
		[0.470]	
Paid Activities (Hours/Week)	33.106***	–1.405	961
	(1.599)	(2.013)	
		[0.470]	
Education Index	–0.129***	–0.083	788
	(0.048)	(0.073)	
		[0.470]	
	PANEL B: AGRICULTURAL INPUTS RECIPIENTS		
	Constant	Respondent preferences respected	N
Monthly Household Income (KES)	7727.768***	3473.461	959
	(910.152)	(4470.320)	
		[0.960]	
Paid Activities (Hours/Week)	34.194***	–0.499	959
	(1.610)	(2.254)	
		[0.970]	
Education Index	–0.058	–0.016	769
	(0.058)	(0.079)	
		[0.970]	
	PANEL C: LIVESTOCK RECIPIENTS		
	Constant	Respondent preferences respected	N
Monthly Household Income (KES)	30545.441	–24237.831	967
	(24384.151)	(24387.862)	
		[0.850]	
Paid Activities (Hours/Week)	30.139***	–0.664	967
	(1.156)	(1.684)	
		[0.850]	
Education Index	0.135***	–0.048	750
	(0.051)	(0.070)	
		[0.850]	

NOTES: Outcome variables are listed on the left. Respondent preferences respected is an indicator that takes the value 1 if either the respondent values the program less than the median respondent and receives cash, or the respondent values the program more than the median respondent and receives the program. Standard errors are reported in parenthesis, FWER-adjusted p-values are reported in brackets. * $p < 0.1$, ** $p < 0.05$, *** $p < 0.01$

Table 37: Effect of cash allocation on secondary outcome variables

	Constant	Cash	N
Monthly Household Income (KES)	1498.682	9124.851	2887
	(4316.169)	(8476.481)	
		[0.570]	
Paid Activities (Hours/Week)	32.097***	0.636	2887
	(1.081)	(1.152)	
		[0.570]	
Education Index	–0.214***	0.087**	2307
	(0.043)	(0.043)	
		[0.050]*	

NOTES: Outcome variables are listed on the left. Regression includes intervention fixed effects with agricultural extension as omitted category. Cash is a dummy which takes the value 1 if respondent received a cash transfer instead of a program. Standard errors are reported in parenthesis, FWER-adjusted p-values are reported in brackets.
* $p < 0.1$, ** $p < 0.05$, *** $p < 0.01$

Table 38: Effect of cash allocation on secondary outcome variables for different intervention recipients

	PANEL A: AGRICULTURAL EXTENSION RECIPIENTS		
	Constant	Cash	N
Monthly Household Income (KES)	6475.075***	−715.300	961
	(714.065)	(849.478)	
		[0.450]	
Paid Activities (Hours/Week)	31.342***	2.130	961
	(1.215)	(2.018)	
		[0.450]	
Education Index	−0.218***	0.097	788
	(0.054)	(0.073)	
		[0.410]	
	PANEL B: AGRICULTURAL INPUTS RECIPIENTS		
	Constant	Cash	N
Monthly Household Income (KES)	7333.572***	4307.926	959
	(832.418)	(4521.255)	
		[0.640]	
Paid Activities (Hours/Week)	33.491***	0.905	959
	(1.491)	(2.254)	
		[0.690]	
Education Index	−0.125**	0.118	769
	(0.056)	(0.079)	
		[0.290]	
	PANEL C: LIVESTOCK RECIPIENTS		
	Constant	Cash	N
Monthly Household Income (KES)	6815.158***	23723.501	967
	(519.977)	(24892.988)	
		[0.960]	
Paid Activities (Hours/Week)	30.353***	−1.079	967
	(1.273)	(1.682)	
		[0.960]	
Education Index	0.086*	0.051	750
	(0.052)	(0.070)	
		[0.960]	

NOTES: Outcome variables are listed on the left. Cash is a dummy which takes the value 1 if respondent received a cash transfer instead of a program. Standard errors are reported in parenthesis, FWER-adjusted p-values are reported in brackets. * $p < 0.1$, ** $p < 0.05$, *** $p < 0.01$

Table 39: Effect of type of program or cash allocation on secondary outcome variables

	(1) Constant	(2) Extension	(3) Cash - Inputs	(4) Inputs	(5) Cash - Livestock	(6) Livestock	(7) Test (p-value): Inputs=Cash	(8) Test (p-value): Livestock=Cash	(9) N
Monthly Household Income (KES)	307.652	715.300	7832.169	3568.753**	30231.008	6507.507*	0.34	0.34	2887
	(3544.939)	(849.624)	(5685.611)	(1694.571)	(25143.157)	(3582.885)			
		[1.000]	[1.000]	[1.000]	[0.290]	[1.000]			
Paid Activities (Hours/Week)	29.070***	–2.130	2.499	1.629	0.203	1.283	0.70	0.52	2887
	(2.673)	(2.018)	(2.554)	(2.461)	(2.890)	(2.961)			
		[0.640]	[0.620]	[0.940]	[0.980]	[1.000]			
Education Index	0.227**	–0.097	–0.012	–0.125	–0.089	–0.140	0.15	0.47	2307
	(0.095)	(0.073)	(0.079)	(0.079)	(0.106)	(0.108)			
		[0.350]	[1.000]	[0.160]	[0.650]	[0.430]			

NOTES: Outcome variables are listed on the left. Regression includes location fixed effects with Kilungu as omitted category. Receiving a cash transfer equivalent to extension is the omitted category. Column (2) Extension refers to respondents randomized to receive the agricutural extension program. Column (3) Cash - Inputs refers to respondents randomized to receive a cash transfer equivalent to inputs. Column (4) Inputs refers to respondents randomized to receive the agricultural inputs program. Column (5) Cash - Livestock refers to respondents randomized to receive a cash transfer equivalent to the livestock package. Column (6) Livestock refers to respondents randomized to receive the livestock package. Column (7) displays the p-value from test for equality of coefficients of inputs and its cash equivalent and column (8) for livestock and its cash equivalent. Standard errors are reported in parenthesis, FWER-adjusted p-values are reported in brackets. * $p < 0.1$, ** $p < 0.05$, *** $p < 0.01$

Table 40: Multivariate regression of secondary outcome variables on indicator for recipient preferences being respected in allocation (as per median valuation) and age

	Constant	Resp pref respected	Age	Resp pref respected X Age	N
Monthly Household Income (KES)	–13649.555	18423.023	562.754	–611.727	2887
	(18519.689)	(14922.022)	(537.692)	(535.676)	
		[0.970]	[0.240]	[0.590]	
Paid Activities (Hours/Week)	34.965***	–0.442	–0.052	–0.002	2887
	(2.904)	(3.598)	(0.065)	(0.082)	
		[0.970]	[0.350]	[0.980]	
Education Index	–0.300***	–0.160	0.003	0.003	2307
	(0.098)	(0.140)	(0.002)	(0.003)	
		[0.590]	[0.240]	[0.590]	

NOTES: Outcome variables are listed on the left. Respondent preferences respected is an indicator that takes the value 1 if either the respondent values the program less than the median respondent and receives cash, or the respondent values the program more than the median respondent and receives the program. Age of respondent is as reported at baseline. Standard errors are reported in parenthesis, FWER-adjusted p-values are reported in brackets. * $p < 0.1$, ** $p < 0.05$, *** $p < 0.01$

Table 41: Multivariate regression of secondary outcome variables on indicator for recipient preferences being respected in allocation (as per cost) and age

	Constant	Resp pref respected	Age	Resp pref respected X Age	N
Monthly Household Income (KES)	–10061.795	13956.361	442.161	–472.195	2887
	(16109.787)	(12407.525)	(457.184)	(465.883)	
		[1.000]	[0.490]	[0.900]	
Paid Activities (Hours/Week)	35.072***	–0.704	–0.051	–0.003	2887
	(2.796)	(3.542)	(0.061)	(0.080)	
		[1.000]	[0.490]	[0.990]	
Education Index	–0.353***	–0.066	0.005**	0.000	2307
	(0.097)	(0.144)	(0.002)	(0.003)	
		[1.000]	[0.010]***	[0.990]	

NOTES: Outcome variables are listed on the left. Respondent preferences respected is an indicator that takes the value 1 if either the respondent values the program less than the cost and receives cash, or the respondent values the program more than the cost and receives the program. Age of respondent is as reported at baseline. Standard errors are reported in parenthesis, FWER-adjusted p-values are reported in brackets. * $p < 0.1$, ** $p < 0.05$, *** $p < 0.01$

Table 42: Multivariate regression of secondary outcome variables on indicator for recipient preferences being respected in allocation (as per median valuation) and gender

	Constant	Resp pref respected	Female	Resp pref respected X Female	N
Monthly Household Income (KES)	24052.468	−22281.628	−22342.020	23458.111	2887
	(18603.516)	(22189.981)	(23143.416)	(22902.585)	
		[0.190]	[0.100]	[0.350]	
Paid Activities (Hours/Week)	36.620***	−1.999	−6.579***	2.514	2887
	(1.988)	(2.212)	(1.882)	(2.555)	
		[0.430]	[0.000]***	[0.530]	
Education Index	−0.144***	−0.027	−0.022	0.008	2307
	(0.055)	(0.072)	(0.062)	(0.089)	
		[0.680]	[0.730]	[0.970]	

NOTES: Outcome variables are listed on the left. Respondent preferences respected is an indicator that takes the value 1 if either the respondent values the program less than the median respondent and receives cash, or the respondent values the program more than the median respondent and receives the program. Female is an indicator for respondent being female. Standard errors are reported in parenthesis, FWER-adjusted p-values are reported in brackets. * $p < 0.1$, ** $p < 0.05$, *** $p < 0.01$

Table 43: Multivariate regression of secondary outcome variables on indicator for recipient preferences being respected in allocation (as per cost) and gender

	Constant	Resp pref respected	Female	Resp pref respected X Female	N
Monthly Household Income (KES)	21479.376	−20243.793	−20611.251	22382.459	2887
	(16439.584)	(20977.130)	(21137.091)	(21277.054)	
		[0.230]	[0.120]	[0.400]	
Paid Activities (Hours/Week)	36.750***	−2.583	−6.663***	2.969	2887
	(1.904)	(2.176)	(1.839)	(2.511)	
		[0.280]	[0.000]***	[0.400]	
Education Index	−0.167***	0.020	0.034	−0.104	2307
	(0.055)	(0.073)	(0.062)	(0.090)	
		[0.830]	[0.680]	[0.400]	

NOTES: Outcome variables are listed on the left. Respondent preferences respected is an indicator that takes the value 1 if either the respondent values the program less than the cost and receives cash, or the respondent values the program more than the cost and receives the program. Female is an indicator for respondent being female. Standard errors are reported in parenthesis, FWER-adjusted p-values are reported in brackets.
* $p < 0.1$, ** $p < 0.05$, *** $p < 0.01$

Table 44: Multivariate regression of secondary outcome variables on indicator for recipient preferences being respected in allocation (as per median valuation) and wealth

	Constant	Resp pref respected	Household wealth	Resp pref respected X Household wealth	N
Monthly Household Income (KES)	10714.932**	–8226.831	–0.000	0.000	2887
	(4861.590)	(8735.392)	(0.000)	(0.000)	
		[0.820]	[1.000]	[1.000]	
Paid Activities (Hours/Week)	32.827***	–0.454	–0.000	–0.000	2887
	(1.283)	(1.190)	(0.000)	(0.000)	
		[0.820]	[1.000]	[1.000]	
Education Index	–0.160***	–0.036	0.000	0.000	2307
	(0.043)	(0.043)	(0.000)	(0.000)	
		[0.820]	[1.000]	[0.220]	

NOTES: Outcome variables are listed on the left. Respondent preferences respected is an indicator that takes the value 1 if either the respondent values the program less than the median respondent and receives cash, or the respondent values the program more than the median respondent and receives the program. Wealth is value of all household assets (in KES) reported at baseline. Standard errors are reported in parenthesis, FWER-adjusted p-values are reported in brackets. * $p < 0.1$, ** $p < 0.05$, *** $p < 0.01$

Table 45: Multivariate regression of secondary outcome variables on indicator for recipient preferences being respected in allocation (as per cost) and wealth

	Constant	Resp pref respected	Household wealth	Resp pref respected X Household wealth	N
Monthly Household Income (KES)	9450.316**	–6665.717	–0.000	0.000	2887
	(4160.175)	(8530.804)	(0.000)	(0.000)	
		[0.910]	[1.000]	[1.000]	
Paid Activities (Hours/Week)	32.920***	–0.374	–0.000	–0.000***	2887
	(1.247)	(1.177)	(0.000)	(0.000)	
		[0.910]	[1.000]	[0.120]	
Education Index	–0.147***	–0.079*	–0.000	0.000**	2307
	(0.041)	(0.044)	(0.000)	(0.000)	
		[0.100]	[1.000]	[0.000]***	

NOTES: Outcome variables are listed on the left. Respondent preferences respected is an indicator that takes the value 1 if either the respondent values the program less than the cost and receives cash, or the respondent values the program more than the cost and receives the program. Wealth is value of all household assets (in KES) reported at baseline. Standard errors are reported in parenthesis, FWER-adjusted p-values are reported in brackets. * $p < 0.1$, ** $p < 0.05$, *** $p < 0.01$

Table 46: Multivariate regression of secondary outcome variables on indicator for recipient preferences being respected in allocation (as per median valuation) and growth mindset

	Constant	Resp pref respected	Growth mindset	Resp pref respected X Growth mindset	N
Monthly Household Income (KES)	40340.947	−33740.334	−926.784	798.357	2887
	(35711.335)	(40058.793)	(965.991)	(983.073)	
		[0.800]	[0.590]	[0.990]	
Paid Activities (Hours/Week)	33.131***	−1.382	−0.012	0.025	2887
	(3.503)	(4.551)	(0.098)	(0.134)	
		[0.830]	[0.900]	[0.990]	
Education Index	−0.246*	−0.120	0.003	0.003	2307
	(0.129)	(0.175)	(0.004)	(0.005)	
		[0.800]	[0.720]	[0.990]	

NOTES: Outcome variables are listed on the left. Respondent preferences respected is an indicator that takes the value 1 if either the respondent values the program less than the median respondent and receives cash, or the respondent values the program more than the median respondent and receives the program. A higher value of the mindset index indicates a growth mindset as opposed to a fixed mindset of the respondent. Standard errors are reported in parenthesis, FWER-adjusted p-values are reported in brackets. * $p < 0.1$, ** $p < 0.05$, *** $p < 0.01$

Table 47: Multivariate regression of secondary outcome variables on indicator for recipient preferences being respected in allocation (as per cost) and growth mindset

	Constant	Resp pref respected	Growth mindset	Resp pref respected X Growth mindset	N
Monthly Household Income (KES)	35606.277	–27698.492	–819.640	655.081	2887
	(31648.319)	(36323.598)	(862.509)	(874.383)	
		[0.950]	[0.770]	[1.000]	
Paid Activities (Hours/Week)	30.311***	4.212	0.078	–0.158	2887
	(3.322)	(4.547)	(0.095)	(0.134)	
		[0.890]	[0.770]	[0.770]	
Education Index	–0.272**	–0.083	0.004	0.001	2307
	(0.124)	(0.176)	(0.004)	(0.005)	
		[0.950]	[0.770]	[1.000]	

NOTES: Outcome variables are listed on the left. Respondent preferences respected is an indicator that takes the value 1 if either the respondent values the program less than the cost and receives cash, or the respondent values the program more than the cost and receives the program. A higher value of the mindset index indicates a growth mindset as opposed to a fixed mindset of the respondent. Standard errors are reported in parenthesis, FWER-adjusted p-values are reported in brackets. * $p < 0.1$, ** $p < 0.05$, *** $p < 0.01$

Table 48: Multivariate regression of secondary outcome variables on indicator for recipient preferences being respected in allocation (as per median valuation) and grit

	Constant	Resp pref respected	Grit	Resp pref respected X Grit	N
Monthly Household Income (KES)	72054.860	−76254.225	−18178.248	20116.060	2887
	(61503.444)	(66134.054)	(16806.431)	(17051.593)	
		[0.330]	[0.240]	[0.470]	
Paid Activities (Hours/Week)	28.658***	−0.791	1.205	0.057	2887
	(5.875)	(7.901)	(1.690)	(2.333)	
		[0.920]	[0.660]	[0.990]	
Education Index	−0.063	−0.110	−0.028	0.026	2307
	(0.205)	(0.288)	(0.058)	(0.084)	
		[0.840]	[0.660]	[0.970]	

NOTES: Outcome variables are listed on the left. Respondent preferences respected is an indicator that takes the value 1 if either the respondent values the program less than the median respondent and receives cash, or the respondent values the program more than the median respondent and receives the program. A higher value of the grit index indicates higher grittiness in respondent's personality. Standard errors are reported in parenthesis, FWER-adjusted p-values are reported in brackets. * $p < 0.1$, ** $p < 0.05$, *** $p < 0.01$

Table 49: Multivariate regression of secondary outcome variables on indicator for recipient preferences being respected in allocation (as per cost) and grit

	Constant	Resp pref respected	Grit	Resp pref respected X Grit	N
Monthly Household Income (KES)	61960.792	−65284.014	−15538.470	17309.135	2887
	(53363.181)	(58225.596)	(14586.593)	(14805.187)	
		[0.360]	[0.370]	[0.480]	
Paid Activities (Hours/Week)	32.958***	−9.798	−0.032	2.617	2887
	(5.669)	(7.875)	(1.636)	(2.327)	
		[0.310]	[1.000]	[0.480]	
Education Index	0.081	−0.424	−0.067	0.110	2307
	(0.201)	(0.289)	(0.057)	(0.085)	
		[0.280]	[0.460]	[0.280]	

NOTES: Outcome variables are listed on the left. Respondent preferences respected is an indicator that takes the value 1 if either the respondent values the program less than the cost and receives cash, or the respondent values the program more than the cost and receives the program. A higher value of the grit index indicates higher grittiness in respondent's personality. Standard errors are reported in parenthesis, FWER-adjusted p-values are reported in brackets. * $p < 0.1$, ** $p < 0.05$, *** $p < 0.01$

Table 50: Multivariate regression of secondary outcome variables on indicator for cash allocation and age

	Constant	Cash	Age	Cash X Age	N
Monthly Household Income (KES)	1528.514	−9352.656	−3.332	421.366	2887
	(4782.925)	(11373.896)	(29.018)	(442.431)	
		[1.000]	[1.000]	[0.960]	
Paid Activities (Hours/Week)	33.617***	2.293	−0.034	−0.038	2887
	(2.516)	(3.552)	(0.054)	(0.080)	
		[1.000]	[0.980]	[0.960]	
Education Index	−0.426***	0.086	0.005**	−0.000	2307
	(0.109)	(0.143)	(0.003)	(0.003)	
		[1.000]	[0.040]**	[0.970]	

NOTES: Outcome variables are listed on the left. Cash is a dummy which takes the value 1 if respondent received a cash transfer instead of a program. Age of respondent is as reported at baseline. Standard errors are reported in parenthesis, FWER-adjusted p-values are reported in brackets. * $p < 0.1$, ** $p < 0.05$, *** $p < 0.01$

Table 51: Multivariate regression of secondary outcome variables on indicator for cash allocation and gender

	Constant	Cash	Female	Cash X Female	N
Monthly Household Income (KES)	1535.885	19965.289	−635.310	−17834.559	2887
	(4747.789)	(21378.504)	(937.901)	(21561.981)	
		[0.230]	[1.000]	[0.540]	
Paid Activities (Hours/Week)	34.125***	2.690	−3.469**	−3.462	2887
	(1.525)	(2.180)	(1.687)	(2.512)	
		[0.240]	[0.090]*	[0.250]	
Education Index	−0.156***	−0.003	−0.084	0.134	2307
	(0.060)	(0.073)	(0.064)	(0.090)	
		[0.940]	[0.160]	[0.250]	

NOTES: Outcome variables are listed on the left. Cash is a dummy which takes the value 1 if respondent received a cash transfer instead of a program. Female is an indicator for respondent being female. Standard errors are reported in parenthesis, FWER-adjusted p-values are reported in brackets. * $p < 0.1$, ** $p < 0.05$, *** $p < 0.01$

Table 52: Multivariate regression of secondary outcome variables on indicator for cash allocation and wealth

	Constant	Cash	Household wealth	Cash X Household wealth	N
Monthly Household Income (KES)	1502.350	9217.061	0.000	−0.000	2887
	(4366.563)	(8618.859)	(0.000)	(0.000)	
		[0.610]	[1.000]	[1.000]	
Paid Activities (Hours/Week)	32.345***	0.429	−0.000**	0.000*	2887
	(1.097)	(1.169)	(0.000)	(0.000)	
		[0.680]	[0.310]	[0.620]	
Education Index	−0.227***	0.102**	0.000*	−0.000*	2307
	(0.044)	(0.043)	(0.000)	(0.000)	
		[0.030]**	[0.050]*	[0.160]	

NOTES: Outcome variables are listed on the left. Cash is a dummy which takes the value 1 if respondent received a cash transfer instead of a program. Wealth is value of all household assets (in KES) reported at baseline. Standard errors are reported in parenthesis, FWER-adjusted p-values are reported in brackets. * $p < 0.1$, ** $p < 0.05$, *** $p < 0.01$

Table 53: Multivariate regression of secondary outcome variables on indicator for cash allocation and growth mindset

	Constant	Cash	Growth mindset	Cash X Growth mindset	N
Monthly Household Income (KES)	–768.003	45830.023	64.607	–1136.757	2887
	(4197.372)	(37983.285)	(63.604)	(916.189)	
		[0.230]	[1.000]	[0.750]	
Paid Activities (Hours/Week)	32.236***	0.329	–0.004	0.010	2887
	(3.303)	(4.551)	(0.094)	(0.134)	
		[0.950]	[1.000]	[0.970]	
Education Index	–0.457***	0.296*	0.008*	–0.007	2307
	(0.133)	(0.176)	(0.004)	(0.005)	
		[0.190]	[0.110]	[0.600]	

NOTES: Outcome variables are listed on the left. Cash is a dummy which takes the value 1 if respondent received a cash transfer instead of a program. A higher value of the mindset index indicates a growth mindset as opposed to a fixed mindset of the respondent. Standard errors are reported in parenthesis, FWER-adjusted p-values are reported in brackets. * $p < 0.1$, ** $p < 0.05$, *** $p < 0.01$

Table 54: Multivariate regression of secondary outcome variables on indicator for cash allocation and grit

	Constant	Cash	Grit	Cash X Grit	N
Monthly Household Income (KES)	−840.439	62249.836	683.244	−15701.520	2887
	(6525.967)	(62011.646)	(1082.560)	(15889.655)	
		[0.390]	[1.000]	[0.580]	
Paid Activities (Hours/Week)	22.113***	12.435	2.924*	−3.456	2887
	(5.542)	(7.870)	(1.638)	(2.325)	
		[0.140]	[0.130]	[0.160]	
Education Index	−0.396*	0.549*	0.053	−0.136	2307
	(0.210)	(0.288)	(0.061)	(0.084)	
		[0.140]	[0.760]	[0.160]	

NOTES: Outcome variables are listed on the left. Cash is a dummy which takes the value 1 if respondent received a cash transfer instead of a program. A higher value of the grit index indicates higher grittiness in respondent's personality. Standard errors are reported in parenthesis, FWER-adjusted p-values are reported in brackets. * $p < 0.1$, ** $p < 0.05$, *** $p < 0.01$

Table 55: Effect on Consumption

	Eq 1: Program X Val-Cost Ratio	Eq 3: R (median val)	Eq 3: R (cost)	Eq 4: Cash
Monthly Per-Capita Cons (KES)	25395.901	−85.747	−140.129	169.366
	(16583.380)	(130.987)	(131.417)	(131.556)
	[0.990]	[0.990]	[0.950]	[0.860]
> Food total (KES)	23298.913***	3.110	−56.624	39.758
	(8006.520)	(66.896)	(67.159)	(67.207)
	[0.860]	[1.000]	[0.970]	[1.000]
» Own food (KES)	12031.257	−209.357*	−116.489	37.395
	(7344.885)	(124.310)	(119.714)	(121.697)
	[1.000]	[0.560]	[0.970]	[1.000]
» Meats bought (KES)	2863.668	4.997	8.392	6.449
	(1763.207)	(14.293)	(13.863)	(13.871)
	[0.990]	[0.990]	[0.990]	[1.000]
» Veg bought (KES)	4802.776**	17.923	−12.743	28.264*
	(2050.015)	(15.059)	(15.483)	(15.500)
	[0.970]	[0.950]	[0.970]	[0.570]
» Cereal bought (KES)	4661.653**	13.523	−24.625	14.785
	(2091.785)	(20.830)	(20.691)	(20.713)
	[0.990]	[0.990]	[0.940]	[1.000]
» Other food bought (KES)	6001.361*	19.402	4.358	18.790
	(3204.280)	(20.998)	(21.866)	(21.897)
	[0.980]	[0.980]	[0.990]	[1.000]
> Temptation goods (KES)	−1396.170	−7.518	9.897	15.474*
	(1682.833)	(9.327)	(9.041)	(9.007)
	[1.000]	[0.990]	[0.950]	[0.640]
> Airtime and internet (KES)	524.907	−4.666	−7.021	11.359*
	(800.132)	(6.536)	(6.429)	(6.423)
	[1.000]	[0.990]	[0.950]	[0.640]
> Transport and hotels (KES)	−869.314	−10.219	−7.852	7.328
	(1665.953)	(8.868)	(8.991)	(9.006)
	[1.000]	[0.950]	[0.970]	[1.000]
> Personal and household items (KES)	−1235.980	−10.924	−5.340	8.537
	(3981.278)	(20.202)	(20.061)	(20.051)
	[1.000]	[0.990]	[0.990]	[1.000]
> Recreation and entertainment (KES)	−699.430	−2.216**	−0.201	1.230
	(592.822)	(1.051)	(0.960)	(0.959)
	[0.080]*	[0.250]	[0.990]	[0.860]
> Housing (KES)	1076.060***	−2.559	2.563	−0.471
	(350.344)	(2.860)	(2.732)	(2.719)
	[0.790]	[0.970]	[0.970]	[1.000]
> Education expenditure (KES)	−967.877	−13.427	−16.495	20.709
	(4841.891)	(26.890)	(26.460)	(26.550)
	[1.000]	[0.990]	[0.990]	[1.000]
> Medical expenditure (KES)	1372.160**	−0.273	−24.152***	26.467***
	(648.660)	(6.359)	(6.340)	(6.372)
	[0.990]	[1.000]	[0.000]***	[0.000]***
> Social expenditure (KES)	181.178	2.717	−9.319*	11.750**
	(644.705)	(4.624)	(4.765)	(4.781)
	[1.000]	[0.990]	[0.300]	[0.140]

NOTES: Outcome variables are listed on the left. Each cell is from a separate regression equation displaying the coefficient for the variable listed in the column header. Standard errors are reported in parenthesis, FWER-adjusted p-values are reported in brackets. * $p < 0.1$, ** $p < 0.05$, *** $p < 0.01$

Table 56: Effect on Food Security

	Eq 1: Program X Val-Cost Ratio	Eq 3: R (median val)	Eq 3: R (cost)	Eq 4: Cash
Food Security Index	4.684	–0.028	–0.041	–0.003
	(5.359)	(0.035)	(0.035)	(0.035)
	[1.000]	[1.000]	[0.550]	[0.960]
> Adults cut/skipped meals	36.123	–0.097	0.357	–0.267
	(29.245)	(0.252)	(0.250)	(0.249)
	[1.000]	[1.000]	[0.520]	[0.780]
> Kids cut/skipped meals	13.142	–0.013	0.215	–0.045
	(23.518)	(0.180)	(0.178)	(0.178)
	[1.000]	[1.000]	[0.550]	[0.960]
> Borrowed food (no. in past month)	470.734	3.134	–3.405	3.424
	(490.099)	(3.123)	(3.392)	(3.427)
	[1.000]	[1.000]	[0.650]	[0.960]
> Household members eat two meals a day (0/1)	8.508**	–0.013	–0.018	0.012
	(3.728)	(0.015)	(0.015)	(0.015)
	[0.140]	[1.000]	[0.550]	[0.960]
> Household members eat until content (0/1)	4.516	–0.016	–0.036**	0.009
	(4.998)	(0.018)	(0.018)	(0.018)
	[0.940]	[1.000]	[0.190]	[0.960]
> Respondent ate eggs meat fish (no. in past week)	–5.471	–0.068	–0.134	–0.301
	(26.481)	(0.543)	(0.536)	(0.538)
	[1.000]	[1.000]	[0.780]	[0.960]

NOTES: Outcome variables are listed on the left. Each cell is from a separate regression equation displaying the coefficient for the variable listed in the column header. Standard errors are reported in parenthesis, FWER-adjusted p-values are reported in brackets. * $p < 0.1$, ** $p < 0.05$, *** $p < 0.01$

Table 57: Effect on Household Income

	Eq 1: Program X Val-Cost Ratio	Eq 3: R (median val)	Eq 3: R (cost)	Eq 4: Cash
Monthly Household Income (KES)	468968.534	−8071.807	−6641.162	9124.851
	(485070.527)	(8521.764)	(8308.534)	(8476.481)
	[1.000]	[0.860]	[0.810]	[0.750]
> Livestock income (KES)	35207.091	−111.466	232.611	−273.636*
	(25001.743)	(156.420)	(156.616)	(156.557)
	[0.910]	[0.900]	[0.370]	[0.250]
> Agricultural income (KES)	562957.606	−7794.364	−6838.254	9454.443
	(478210.751)	(8499.965)	(8286.808)	(8454.506)
	[1.000]	[0.880]	[0.780]	[0.720]
> Enterprise income (KES)	−4143.023	−19.605	−130.257	38.085
	(5077.370)	(96.219)	(92.987)	(93.740)
	[1.000]	[0.900]	[0.370]	[0.900]
> Wage income (KES)	−120910.118**	−126.768	224.995	−132.125
	(53985.174)	(251.845)	(248.541)	(248.155)
	[0.160]	[0.900]	[0.700]	[0.900]

NOTES: Outcome variables are listed on the left. Each cell is from a separate regression equation displaying the coefficient for the variable listed in the column header. Standard errors are reported in parenthesis, FWER-adjusted p-values are reported in brackets. * $p < 0.1$, ** $p < 0.05$, *** $p < 0.01$

Table 58: Effect on Household Assets

	Eq 1: Program X Val-Cost Ratio	Eq 3: R (median val)	Eq 3: R (cost)	Eq 4: Cash
Household Assets (KES)	309395.643	–821.294	–423.570	–321.023
	(278921.710)	(1185.293)	(1174.823)	(1174.576)
	[0.830]	[0.950]	[0.980]	[0.920]
> Value of Productive Assets (KES)	–63184.932	–138.734	–244.540	302.245*
	(47574.080)	(179.872)	(176.695)	(176.880)
	[0.650]	[0.940]	[0.620]	[0.360]
> Value of Vehicles (KES)	–13157.186	85.469	450.019	–419.024
	(63618.711)	(358.984)	(361.323)	(360.259)
	[0.980]	[0.990]	[0.660]	[0.750]
> Value of Furniture (KES)	13613.834	–39.689	–381.071	255.661
	(99466.527)	(384.465)	(384.122)	(384.378)
	[0.980]	[0.990]	[0.750]	[0.920]
> Value of Household Durables (KES)	27571.351	21.334	–48.293	131.486
	(38954.499)	(181.243)	(180.122)	(180.270)
	[0.940]	[0.990]	[0.980]	[0.920]
> Value of Livestock (KES)	187067.045	–548.294	–70.489	–354.530
	(206884.151)	(679.414)	(672.308)	(672.216)
	[0.830]	[0.940]	[0.980]	[0.920]
> Value of Financial Assets (KES)	157485.544	–201.381	–129.196	–236.860
	(135095.477)	(389.777)	(381.581)	(380.542)
	[0.510]	[0.990]	[0.980]	[0.920]

NOTES: Outcome variables are listed on the left. Each cell is from a separate regression equation displaying the coefficient for the variable listed in the column header. Standard errors are reported in parenthesis, FWER-adjusted p-values are reported in brackets. * $p < 0.1$, ** $p < 0.05$, *** $p < 0.01$

Table 59: Effect on Psychological Indices

	Eq 1: Program X Val-Cost Ratio	Eq 3: R (median val)	Eq 3: R (cost)	Eq 4: Cash
Psychological Wellbeing Index	20.575***	0.013	−0.067*	0.059
	(7.049)	(0.037)	(0.036)	(0.036)
	[0.220]	[0.970]	[0.350]	[0.360]
> CESD (depression)	−118.115*	0.037	0.523	−0.295
	(67.515)	(0.336)	(0.333)	(0.333)
	[0.410]	[0.970]	[0.500]	[0.840]
> GHQ12 (not feeling useful)	−1.803	−0.010	−0.068**	0.057*
	(6.477)	(0.031)	(0.030)	(0.030)
	[0.850]	[0.970]	[0.200]	[0.260]
> GHQ12 (incapable of making decisions)	6.567	−0.023	−0.006	0.007
	(7.950)	(0.028)	(0.028)	(0.028)
	[0.590]	[0.910]	[0.970]	[0.960]
> GHQ12 (could not overcome difficulties)	−12.622*	−0.034	0.014	−0.024
	(6.493)	(0.031)	(0.031)	(0.031)
	[0.360]	[0.840]	[0.970]	[0.840]
> WVS (unhappiness)	−14.485**	−0.017	0.014	0.002
	(6.688)	(0.027)	(0.027)	(0.027)
	[0.240]	[0.950]	[0.970]	[0.960]
> WVS (life dissatisfaction)	−43.455***	−0.058	0.114	−0.133*
	(12.726)	(0.081)	(0.080)	(0.080)
	[0.240]	[0.950]	[0.500]	[0.360]

NOTES: Outcome variables are listed on the left. Each cell is from a separate regression equation displaying the coefficient for the variable listed in the column header. Standard errors are reported in parenthesis, FWER-adjusted p-values are reported in brackets. * $p < 0.1$, ** $p < 0.05$, *** $p < 0.01$

Table 60: Effect on Autonomy

	Eq 1: Program X Val-Cost Ratio	Eq 3: R (median val)	Eq 3: R (cost)	Eq 4: Cash
Autonomy Index	3.973	−0.055	−0.111***	0.125***
	(9.573)	(0.038)	(0.038)	(0.038)
	[1.000]	[0.840]	[0.040]**	[0.000]***
> I make important decisions in my life for myself	8.647	−0.009	−0.020	−0.014
	(6.199)	(0.022)	(0.021)	(0.021)
	[0.780]	[1.000]	[0.930]	[0.970]
> Other people and orgs enable me to live with dignity	15.154*	0.006	0.008	−0.022
	(8.913)	(0.035)	(0.035)	(0.035)
	[0.670]	[1.000]	[0.980]	[0.970]
> NGOs trust the people they seek to help	0.251	−0.012	−0.054**	0.065**
	(8.670)	(0.026)	(0.026)	(0.026)
	[1.000]	[1.000]	[0.260]	[0.110]
> I would rather have little money but make my own decisions	14.687*	−0.004	−0.061**	0.026
	(7.714)	(0.025)	(0.024)	(0.024)
	[0.240]	[1.000]	[0.090]*	[0.890]
> Org from whom I received aid treated me as an equal	−8.129	−0.022	−0.029	0.025
	(6.446)	(0.027)	(0.027)	(0.027)
	[0.940]	[1.000]	[0.870]	[0.900]
> Org from whom I received aid treated me with contempt	1.566	−0.017	0.116***	−0.111***
	(13.015)	(0.039)	(0.038)	(0.038)
	[1.000]	[1.000]	[0.030]**	[0.020]**
> Org from whom I received aid was arrogant	−0.540	−0.011	0.024	−0.006
	(8.149)	(0.020)	(0.021)	(0.021)
	[1.000]	[1.000]	[0.820]	[0.970]
> Aid was tailored to solve my problems	−4.723	−0.049	−0.146***	0.250***
	(9.657)	(0.032)	(0.032)	(0.032)
	[1.000]	[0.800]	[0.000]***	[0.000]***
> Org from whom I received aid treated me as an individual	−7.089	−0.019	−0.165***	0.247***
	(7.272)	(0.032)	(0.032)	(0.032)
	[0.990]	[1.000]	[0.000]***	[0.000]***
> Org from whom I received aid ridiculed me	0.671	0.006**	0.002	−0.004
	(0.439)	(0.003)	(0.003)	(0.003)
	[0.990]	[0.460]	[0.930]	[0.800]
> I felt that I could ask the org for what I needed	1.357	−0.006	0.037**	−0.064***
	(5.723)	(0.019)	(0.019)	(0.019)
	[1.000]	[1.000]	[0.270]	[0.000]***
> Org from whom I received aid reduced my sense of control	4.017	0.008	−0.004	0.004
	(5.569)	(0.016)	(0.016)	(0.016)
	[0.990]	[1.000]	[0.980]	[0.970]
> Org tried to persuade me to make a particular decision	5.189	0.015	0.034**	−0.038***
	(3.455)	(0.015)	(0.015)	(0.015)
	[0.930]	[0.970]	[0.140]	[0.070]*
> Org made me feel in control of my life	6.859	0.003	−0.003	0.020
	(5.168)	(0.016)	(0.016)	(0.016)
	[0.860]	[1.000]	[0.980]	[0.830]

NOTES: Outcome variables are listed on the left. Each cell is from a separate regression equation displaying the coefficient for the variable listed in the column header. Standard errors are reported in parenthesis, FWER-adjusted p-values are reported in brackets. * $p < 0.1$, ** $p < 0.05$, *** $p < 0.01$

Table 61: Effect on Labour

	Eq 1: Program X Val-Cost Ratio	Eq 3: R (median val)	Eq 3: R (cost)	Eq 4: Cash
Paid Activities (Hours/Week)	−202.179	−0.566	−0.857	0.636
	(246.865)	(1.159)	(1.151)	(1.152)
	[0.800]	[0.960]	[0.800]	[0.860]
> Agricultural activities (Hours/Week)	−5.087	−0.131	−0.434	0.181
	(141.754)	(0.552)	(0.554)	(0.555)
	[0.970]	[0.980]	[0.800]	[0.900]
> Tending animals (Hours/Week)	26.671	0.011	−0.207	0.306
	(62.513)	(0.331)	(0.332)	(0.332)
	[0.970]	[1.000]	[0.800]	[0.800]
> Non-farm/livestock business (Hours/Week)	−69.837	−0.629**	−0.302	0.245
	(56.215)	(0.286)	(0.283)	(0.283)
	[0.720]	[0.070]*	[0.710]	[0.800]
> Paid work outside home (Hours/Week)	−153.926	0.183	0.086	−0.095
	(128.107)	(0.508)	(0.504)	(0.503)
	[0.600]	[0.970]	[0.890]	[0.900]

NOTES: Outcome variables are listed on the left. Each cell is from a separate regression equation displaying the coefficient for the variable listed in the column header. Standard errors are reported in parenthesis, FWER-adjusted p-values are reported in brackets. * $p < 0.1$, ** $p < 0.05$, *** $p < 0.01$

Table 62: Effect on Education

	Eq 1: Program X Val-Cost Ratio	Eq 3: R (median val)	Eq 3: R (cost)	Eq 4: Cash
Education Index	28.862***	−0.022	−0.050	0.087**
	(9.190)	(0.043)	(0.043)	(0.043)
	[0.060]*	[0.990]	[0.710]	[0.120]
> Proportion children in school	2.230	−0.005	−0.005	0.015
	(2.197)	(0.011)	(0.011)	(0.011)
	[0.710]	[0.990]	[1.000]	[0.440]
> Avg. school days misssed (neg coded)	59.765***	−0.044	−0.049	0.125
	(22.811)	(0.120)	(0.120)	(0.120)
	[0.170]	[0.990]	[1.000]	[0.650]
> Avg. perceived school performance	0.139	0.010	−0.002	0.022
	(4.427)	(0.016)	(0.016)	(0.016)
	[0.950]	[0.970]	[1.000]	[0.440]
> Avg. school spending per child (KES)	202915.793**	−434.108	−124.771	235.424
	(101539.396)	(526.610)	(520.453)	(521.213)
	[0.320]	[0.970]	[1.000]	[0.680]
> Avg. expected highest level of education per child	84.267*	−0.085	−0.389**	0.381**
	(49.183)	(0.159)	(0.158)	(0.158)
	[0.150]	[0.990]	[0.080]*	[0.050]*
> Avg. time studying per child (last week)	120.464	0.105	−0.127	−0.689
	(183.082)	(0.863)	(0.859)	(0.859)
	[0.770]	[0.990]	[1.000]	[0.650]

NOTES: Outcome variables are listed on the left. Each cell is from a separate regression equation displaying the coefficient for the variable listed in the column header. Standard errors are reported in parenthesis, FWER-adjusted p-values are reported in brackets. * $p < 0.1$, ** $p < 0.05$, *** $p < 0.01$
